# A coupled model to value indirect impacts of maternal, newborn and child health interventions

**DOI:** 10.1371/journal.pgph.0005507

**Published:** 2026-11-06

**Authors:** Tom Walsh, Joshua P. Vogel, Maureen Makama, Romesh Abeysuriya, Nick Scott

**Affiliations:** 1 Burnet Institute, Melbourne, Australia; 2 School of Public Health and Preventive Medicine, Monash University, Melbourne, Australia; Aga Khan University, PAKISTAN

## Abstract

Outcomes associated with maternal, newborn and child health (MNCH) conditions have improved significantly over the past 20 years, however, they are not on track to meet the global SDG targets by 2030. Emerging interventions have the potential to greatly increase access to care, but estimating their impacts is difficult due to complicated interactions between interventions. Models exist to quantify outcomes and costs associated with scaling up interventions related to MNCH (e.g., The Lives saved tool (LiST)), but none have been designed to flexibly integrate novel interventions. This study aimed to develop a model that can estimate the impact of standard-of-care (SOC) and novel interventions on MNCH conditions, and present an example use case. We developed a compartment-based model with 15 interconnected population- and condition-specific modules for pregnant women (in different trimesters), newborns and children <5 years to quantify impacts of bespoke novel interventions. The model included modules for anemia, pre-eclampsia, obstructed labour, hemorrhage, maternal sepsis, preterm, small-for-gestation-age (SGA), respiratory distress syndrome (RDS), neonatal sepsis, birth asphyxia, wasting (neonates and children), stunting and modules that captures neonatal and childhood conditions not captured elsewhere in the model. The model captures dynamic interactions between MNCH conditions, including across trimesters, and accounts for the full impact of early detection and prevention of conditions during pregnancy. In an example use case, the model identified that scaling-up standard-of-care and novel diagnostic interventions for anemia and pre-eclampsia plus AI-ultrasound (for early detection of obstructed labour, hemorrhage and birth outcome risks, leading to facility referrals) to 60% could avert 207 million DALYs globally over 2024–2040. We have developed a modelling tool which can be flexibly adapted to assess the impacts of novel interventions for MNCH conditions, and can be applied at a global, regional, national or sub-national level.

## Introduction

Each day there are approximately 500 maternal deaths and 5,000 neonatal deaths globally [1]. Approximately 95% of these deaths occur in low-and middle-income countries (LMICs), and most are a result of preventable or treatable conditions. In 2015, the Sustainable Development Goals (SDG) initiative set targets for neonatal and maternal mortality to be reduced to below 12 per 1,000 live births and 70 per 100,000 live births by 2030, respectively [2]. While maternal, newborn and child health (MNCH) outcomes have drastically improved throughout much of the world over the last two decades, progress in many LMICs stalled during the COVID-19 pandemic. Significant acceleration of progress will be required to reach global and national SDG targets for maternal and newborn deaths by 2030.

Mathematical models can simulate interactions between MNCH conditions. They are a useful tool to estimate the relative benefits of scaling up different packages of interventions. Models already exist which quantify the impact of interventions on MNCH conditions - the most widely used being the lives saved tool (LiST) [3]. LiST is an industry standard modelling tool that has been used in many analyses to inform public policy surrounding investment into MNCH. However, there are unique challenges associated with modelling MNCH interventions that limit the utility of existing tools.

MNCH conditions have complex interactions, such that preventing or treating one condition can often have impacts on the incidence of other conditions. For example, maternal anemia is a risk factor for maternal mortality [4,5], being small-for-gestational-age (SGA) [6] and for being born preterm [7]. SGA and preterm birth are themselves risk factors for neonatal mortality [8], stunting and wasting [9]. Therefore, population-level initiatives to prevent anaemia could reduce the prevalence of multiple other ‘downstream’ conditions. These dynamics are important to include, in order to capture the full direct and downstream benefits of introducing MNCH interventions.

An additional complexity is that to effectively treat or manage many of these conditions, diagnosis and clinical monitoring interventions are required. These may not always be available, particularly in LMICs. For example, the upfront cost of antenatal ultrasound devices, as well as shortages of healthcare professionals who can use them accurately, prevents conditions such as fetal growth restriction from being detected and hence limits the impact that subsequent interventions can have. Similarly, pre-eclampsia is a leading cause of maternal mortality, yet diagnosis relies on accurate measurement of blood pressure, proteinuria and other forms of organ dysfunction, which may not always be available. In the case of pre-eclampsia, there is a need for new risk screening and diagnostic tools to expand coverage, and there are pipelines of such products coming to market [10]. Quantifying the impacts of scaling up current MNCH interventions, and what additional value novel interventions may bring, helps to prioritize investments in health and research programs. While some other models, including LiST, capture interactions between interventions, risk factors and outcomes, they are not able to flexibly integrate new interventions, or consider more complex interactions.

To address this, we developed a model in collaboration with the Bill and Melinda Gates Foundation (BMGF), which captures the complex interactions between interventions, risk factors and outcomes and also enables flexible integration of new interventions. The model enables scenarios to be projected, where combinations of emerging technologies and existing interventions are implemented and scaled up. This allows the user to identify packages with the greatest potential for impact. In this paper, we describe the key aspects of the model design and demonstrate an example use case.

## Methods

The model outlined in this study was developed around a set of interventions included in a study commissioned by and conducted for the BMGF. This analysis implements a subset of these interventions. The interventions under consideration in the BMGF study informed the structure and guided the model decisions.

### Model overview and study design

This study developed a mathematical compartmental model and used it to compare model projections for a continued status quo scenario to scenarios with scaled-up coverages for a set of diagnostic and novel interventions. The model was implemented using the Atomica modeling framework (version 1.32.0) [11]. Atomica was selected for its ability to flexibly modify model structure and add new interconnected modules. Framework and databook files for the model presented here are provided in S1 Appendix. Fifteen distinct, interconnected MNCH modules were constructed to account for the intergenerational linkages between maternal, fetal, newborn and infant risk factors and outcomes (Fig 1, Table 1). These linkages are based on evidence from literature and are described in detail in Table B in S1 Appendix. Each module is a deterministic compartmental model, which is linked to one or more other modules through shared parameters (e.g., the prevalence of maternal anemia is an output of the anemia module, and an input parameter for the birth outcome module).

**Table 1:** Key aspects of modules within the framework.

Timestep	Module	Description	Inputs from other modules	Outputs for a given intervention scenario
Pregnancy (three distinct trimesters modelled)	ANC	Tracks ANC attendance through pregnancy and allows ultrasound coverage to be scaled up Tracks referral facility attendance for childbirth. Referral facility attendance is a requirement for a number of childbirth interventions and reduces the impact of conditions that occur at childbirth.	Number of pregnant women	Proportion attending ANC Referral facility Ultrasound coverage Number of pregnant women that continue to childbirth
	Anemia (Intrapartum and postpartum)	Tracks anemia status in pregnant women, split into mild, moderate and severe.	Number of pregnant women Proportion attending ANC Hemorrhage prevalence (postpartum anemia)	Number of pregnant women with anemia (3rd trimester and postpartum) Number of DALYs due to anemia (during pregnancy and postpartum)
	Pre-eclampsia	Tracks preeclampsia in pregnant women with early onset and late onset pre-eclampsia and the impact of screening and treatment interventions.	Number of pregnant women Proportion attending ANC	Number of morbidities, mortalities and DALYs due to pre-eclampsia (for each trimester) Prevalence of early and late onset pre-eclampsia
Childbirth	Obstructed labour	Tracks obstructed labour in childbirth. Obstructed labour can be averted by a medically necessary Caesarean section.	Number of pregnant women that continue to childbirth Proportion with RF delivery Ultrasound coverage (Necessary c-section identification)	Number of morbidities, mortalities and DALYs due to obstructed labour Prevalence of obstructed labour (with and without c-section)
	Postpartum hemorrhage	Tracks postpartum hemorrhage in pregnant women during the modelled childbirth timestep.	Number of pregnant women that continue to childbirth Proportion with RF delivery Ultrasound coverage (identification of likely hemorrhage for referral to higher level facility) Anemia prevalence	Number of morbidities, mortalities and DALYs due to hemorrhage
	Maternal nutrition interventions	Tracks coverage of maternal nutrition interventions in pregnant women.	Number of pregnant women that continue to childbirth	Coverage of maternal nutrition interventions (i.e., MMS, IFAS)
	Maternal sepsis	Tracks sepsis in women during childbirth.	Number of pregnant women that continue to childbirth	Number of morbidities, mortalities and DALYs due to maternal sepsis Prevalence of maternal sepsis
	Preterm/SGA	Tracks preterm and/or SGA birth outcomes. Associated morbidities can be reduced in severity by delivery at a referral facility. Facility delivery for imminent preterm and/or SGA births can be increased by accurate detection with ultrasound.	Number of pregnant women that continue to childbirth Prevalence of maternal anemia (3rd trimester) Prevalence of preeclampsia	Number of morbidities, mortalities and DALYs due to preterm and/or SGA Prevalence of preterm and/or SGA
Neonatal time period	RDS	Tracks birth asphyxia and subsequent neonatal encephalopathy in neonates during the neonatal period and calculates number of births which are stillbirth during pregnancy.	Number of pregnant women that continue to childbirth Prevalence of obstructed labour Prevalence of obstructed labour without c-section Prevalence of maternal sepsis Coverage of maternal nutrition interventions	Number of live births Number of morbidities, mortalities and DALYs due to neonatal encephalopathy Number of and DALYs due to stillbirth
	RDS	Tracks RDS in preterm neonates during the neonatal period. Any prevention can only be targeted towards those who are identified as preterm.	Number of live births Prevalence of preterm births	Number of morbidities, mortalities and DALYs due to RDS
	Neonatal sepsis	Tracks neonatal sepsis in neonates during the neonatal period.	Number of live births Prevalence of preterm births	Number of morbidities, mortalities and DALYs due to neonatal sepsis
	Stunting	Tracks stunting risk during the neonatal time period, and associated disability due to stunting.	Number of live births Coverage of maternal nutrition interventions Prevalence of preterm/SGA birth outcomes	Number of morbidities, mortalities and DALYs due to stunting
	Other neonatal conditions (encephalitis, meningitis, enteric infections, respiratory infections, and malaria)	Tracks neonatal conditions otherwise not captured in the model during the neonatal period.	Number of li ve births	Number of morbidities, mortalities and DALYs due to other neonatal conditions
Infant - childhood time period	Wasting	Tracks wasting in infants and children from 8 days to 5 years of life. Each cohort of neonates in the 3 month time period are tracked for the 5 years with the timesteps split into 8 days - 5 months, 6 months - 18 months and 19 months to 59 months.	Number of surviving neonates Prevalence of preterm/SGA birth outcomes	Number of morbidities, mortalities and DALYs due to wasting

**Fig 1:**
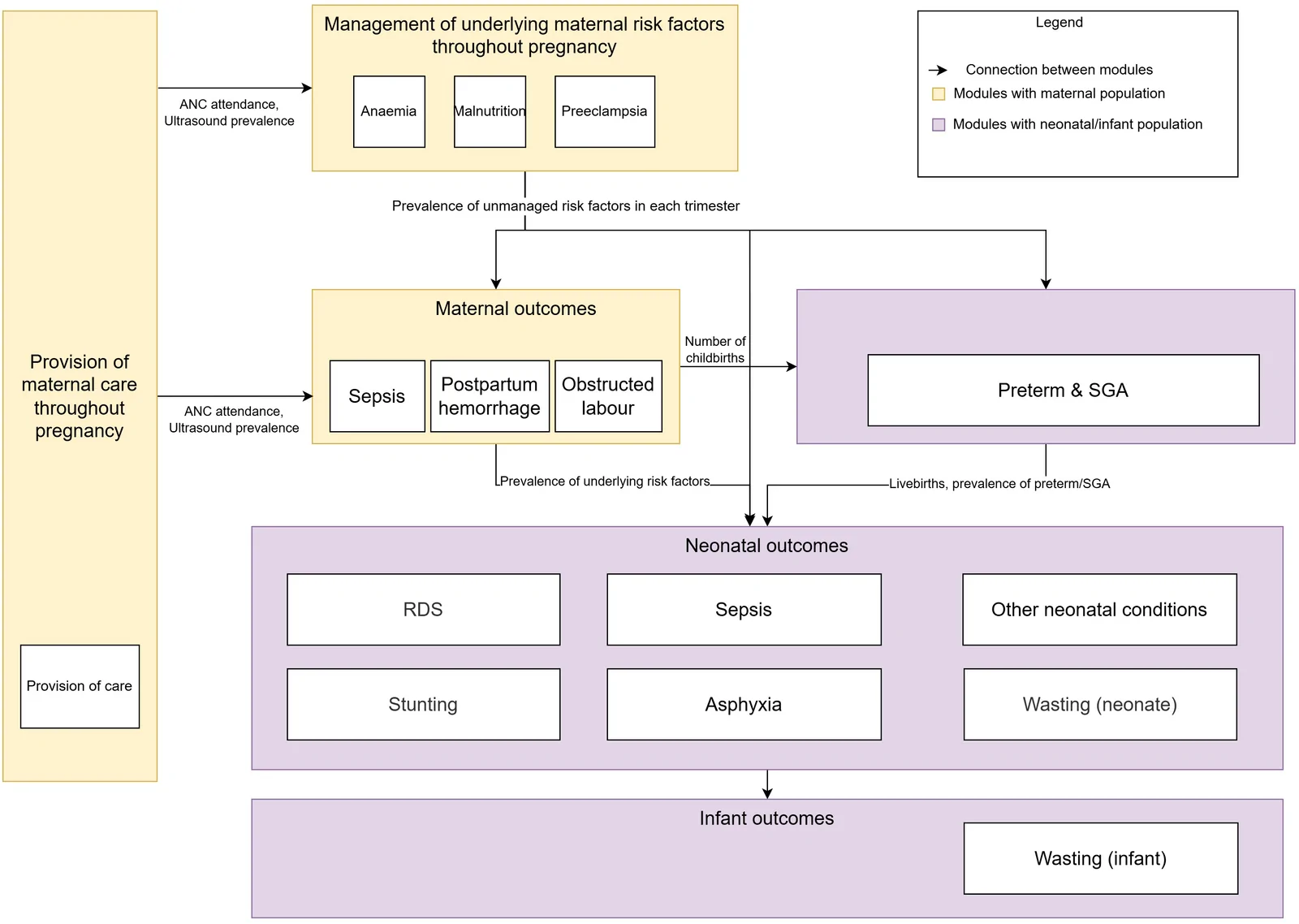
Model structure for capturing morbidities and mortalities for specific conditions, highlighting interactions between conditions.

Women enter the model population when they become pregnant and are tracked across trimesters, with childbirth and immediate postpartum outcomes recorded. Within each trimester, fetal development is also modelled. The model captures the prevalence of different birth outcomes (pre-term and/or small for gestational age [SGA]) and tracks newborn and infant outcomes up to age five. By explicitly modelling the three trimesters for pregnant women, the model can account for the impact of early intervention during pregnancy.

The model produces outputs for the prevalence of a range of MNCH outcomes, morbidities and mortality, which are converted to disability-adjusted life years (DALYs). The 15 modules are split into 6 distinct sections as shown in Fig 1 and as described below.

The choice of pathways for the different modules and the connections between modules were developed in consultation with content experts at the Gates Foundation, who contributed to model development, sourcing of parameters, and expert opinion on intervention effect sizes where published evidence was limited. The resulting pathways were confirmed to be consistent with those used in other established MNCH models, such as LiST, by content experts., by content experts [3]..

#### Provision of care.

The provision of care module tracks the coverage of antenatal care during each trimester, which influences the maximum achievable coverage of other interventions, as well as referrals to differently equipped facilities for birth (Fig A in S1 Appendix).

#### Maternal risk factors.

Maternal risk factors that are modelled include anemia, pre-eclampsia and undernutrition. The maternal anemia and pre-eclampsia modules track the development, detection and management of these conditions across trimesters, while the maternal nutrition module tracks the prevalence and treatment of undernutrition during pregnancy. Outputs of these modules are the prevalence of maternal anemia (mild, moderate or severe), prevalence of preeclampsia (treated, untreated), pre-eclampsia-related mortality, and the prevalence of maternal undernutrition. These outputs are themselves inputs for the maternal outcome, birth outcome, and newborn outcome sections.

#### Maternal outcomes.

Maternal outcomes include peripartum sepsis, postpartum hemorrhage and obstructed labour occurring around the time of childbirth, and their prevalence is dependent on outputs of the provision of care and maternal risk factor modules. Outputs include the prevalence of morbidities and mortality due to each condition. These outputs are themselves risk factors for the neonatal modules.

#### Birth outcomes.

The birth outcome timestep also tracks the prevalence of preterm and SGA births, as well as associated morbidities and mortalities. This module is dependent on outputs from the provision of care and maternal risk factor modules, and the prevalence of preterm and/or SGA birth influence the neonatal outcomes module.

#### Neonatal outcomes.

Neonatal outcomes that are modelled include respiratory distress syndrome (RDS), sepsis, stunting, asphyxia, wasting, other neonatal conditions. Other neonatal conditions cover mortalities related to neonatal conditions that are not captured elsewhere in this model. The modules track the development, detection and management of these conditions in the neonatal period (up to 28 days postpartum). Outcomes of these modules are the prevalence of RDS, sepsis, stunting, asphyxia, wasting and other neonatal conditions, and mortality due to RDS, sepsis, neonatal encephalopathy, wasting and other neonatal conditions. These outcomes are themselves inputs for the infant outcome modules.

#### Infant and child outcomes.

The infant and child outcome that is modelled is wasting in the infant and childhood time period. The modules track the development, detection and management of wasting in the infant period up to 5 years post birth. Outcomes include morbidities and mortalities due to wasting.

#### Modules.

Key aspects of each module within the framework are described in Table 1.

### Model parameters

The model requires setting-specific inputs for demographics, prevalence of MNCH conditions, and maternal and newborn mortality rates. Parameters are also required for estimated relative risks between conditions, as well as baseline intervention and health system coverage. For many settings, the required data can be obtained from the Institute for Health Metrics and Evaluation (IHME) [12] and relative risks are available from systematic reviews and meta-analyses (Tables A, B and C in S1 Appendix).

### Model outputs

Health benefits that are estimated when the coverage of interventions in the model increases include cases of anemia, PPH, obstructed labour, maternal sepsis, preterm, SGA, neonatal encephalopathy, wasting, stunting, neonatal sepsis averted and maternal deaths, stillbirths and newborn deaths averted. This analysis takes a health-outcomes perspective: it quantifies morbidity, mortality and DALYs averted under each scenario, and does not incorporate costs, healthcare system resource use, or a cost-effectiveness framework.

Additionally, the model calculates the total DALYs and DALYs averted for each scenario. This model contrasts to previous models in that it aims to explicitly account for intervention impacts via direct intervention impact on specific conditions, and subsequent indirect impacts on other, downstream conditions. In such a way we attempt to both recategorize previously captured impacts and account for previously uncaptured impacts.

### Interventions

Model outputs can be impacted by interventions. To be included in the model, an intervention requires one or more target modules, a target parameter within those modules, effect size on the parameter, baseline coverage, and maximum achievable coverage. For example, standard-of-care (SOC) iron tablets during pregnancy target the anemia module. It has an estimated relative reduction of 95% for severe, 88% for moderate and 76% for mild anemia and a maximum achievable coverage of 95% based on the proportion who attend ANC.

Changes in intervention coverage therefore cause changes in outcomes of the target modules (“direct effects”), as well as changes in downstream outcomes (“indirect effects”). The attributes for an example setting are described in Table 2.

**Table 2:** Summary of direct and indirect impacts of each interventions used in the example application.

Intervention	Direct impacts	Indirect impacts	Eligible population	Effect size (relative risk unless otherwise specified)	Data source
Ready-to-use therapeutic foods	Reduce wasting		Children with wasting (hospitalized or not hospitalized) for 6 to 18 months and 19–59 months	Severe: 0.58 Moderate: 0.36	Isanaka et al. JAMA 2009 [15].
Iron folic acid tablets	Reduces anemia	Reduces hemorrhage Reduces postpartum anemia	Identified first/second trimester mild/moderate/severe anemia attending ANC	Severe treatment: 0.95 Moderate treatment: 0.88 Mild treatment: 0.76	Oh C, et al. Nutrients. 2020 [16]
Pre-eclampsia urine dipstick test	Detects pre-eclampsia of all severity	Reduces stillbirth	Women who will experience second/third trimester preeclampsia attending ANC	Sensitivity = 0.68 (Value is for women with suspected preeclampsia but is a reasonable estimate for the modelled population.)	Teeuw HM, et al. Diagnostic accuracy of urine dipstick tests for proteinuria in pregnant women suspected of preeclampsia: A systematic review and meta-analysis. Pregnancy Hypertens. 2022 Mar;27:123–130 [17].
SOC Ultrasound	Identifies risk factors for obstructed labor Identifies risk factors for hemorrhage Estimates gestational age allowing more accurate identification of preterm birth and SGA	Reduces adverse outcomes associated with Hemorrhage, Obstructed labour and Preterm/SGA Reduces stillbirth due to reduction in adverse effects of obstructed labour Reduces neonatal encephalopathy/ RDS adverse outcomes due to referral facility birth	All women ever attending ANC (Maximum 50% coverage)	Identification of risk factors for • Obstructed labor: 0.9 (Assuming all cases require c-section) • Hemorrhage: 0.57 • Preterm: 0.5 • SGA: 0.6	Estimates from primary data from VITAL Pakistan Pregnancy Cohort. Ultrasound-identifiable risk factor for hemorrhage defined as presence of multiple, placenta previa, or breech presentation (breech is the only type of malpresentation measured in the database but this is the most common type.)
Pre-eclampsia treatment (severe PE only)	Reduces impacts of severe pre-eclampsia	Reduces: • non-RDS preterm • SGA/ • RDS • Stillbirth due to reduced pre-eclampsia	Women with identified severe PE attending ANC	Maternal morbidity: 0.6 Maternal mortality: 0.6	Duley et al. Cochrane Database Syst Rev. 2010 [14]
Hb test	Diagnoses anemia	Reduces anemia by increasing treatment Reduces postpartum hemorrhage Reduces: • Non- RDS preterm • SGA • Stunting • Wasting due to reduced anemia	All pregnant women (first/second trimester) attending ANC	Sensitivity = 0.667	Assumed value based on HemoCue system: 1. Fothergill et al. J Nutr. 2023 [18] 2. Alarcón-Yaquetto et al. PLoS One. 2023 [19] 3. Sanchis-Gomar et al. J Lab Autom 2013 [20]
Non-invasive Hb test	Diagnoses anemia	Reduces anemia by increasing treatment Reduces hemorrhage Reduces Non- RDS preterm/SGA/Stunting/Wasting due to reduced anemia	All pregnant women (first/second trimester) attending ANC	Sensitivity = 0.95	Assumption based on expert opinion, using a ‘plausible’ maximum for sensitivity
Pre-eclampsia risk screening and prevention	Identifies and reduces likelihood of pre-eclampsia occurring	Reduces Non- RDS preterm/SGA/ RDS /Stillbirth due to reduced pre-eclampsia	Women at high risk of PE attending first trimester ANC	Early onset PE incidence: 0.8	Assumption consistent with Duley et al. 2019 Cochrane (18% reduction)
AI ultrasound	Identifies risk factors for obstructed labor Identifies risk factors for hemorrhage Estimates gestational age allowing more accurate identification of preterm birth and SGA Increases coverage of ultrasound compared to SOC ultrasound	Reduces adverse outcomes associated with Hemorrhage, Obstructed labour and Preterm/SGA Reduces stillbirth due to reduction in adverse effects of obstructed labour Reduces neonatal encephalopathy/RDS adverse outcomes due to referral facility birth	All women ever attending ANC (note: intervention increases coverage of this population)	Identification of risk factors for • Obstructed labor: 0.9 • Hemorrhage: 0.57 • Preterm: 0.5 • SGA: 0.6 Note: main impact is higher coverage for AI ultrasound due to accessibility Note: other impacts are captured through links between conditions	Same values as for SOC ultrasound

In the model, both standard-of-care and novel interventions modify the prevalence and outcomes of conditions. Interventions can be flexibly introduced and removed on a per-analysis basis.

### Model calibration

The model is calibrated such that when it is run with baseline intervention coverage, it reproduces the burden of maternal anemia, preeclampsia, maternal sepsis, postpartum hemorrhage, obstructed labour, preterm births, SGA births, neonatal deaths (by cause), stunting, wasting and infant deaths (by cause). This is achieved by varying the probability of developing these conditions in each module. It is assumed that a single year captures the relationship between conditions within the model. This calibration occurs with SOC interventions included in the model. Prior to calibration, each module was checked for expected behavior at parameter extremes — for example, confirming that condition-specific prevalence approached zero under 100% treatment coverage, and that removing an intervention returned outputs to baseline values. Module-level baseline outputs (e.g., prevalence of anemia, pre-eclampsia, and preterm/SGA birth) were also compared against IHME GBD estimates independently of the calibration procedure, to confirm consistency with observed burden prior to model calibration.

### Example application

We describe an example use case of the model based on a recent study by the BMGF examining a set of standard of care and novel interventions under development [13], which used an earlier version of this model. For this analysis we considered two coverage scenarios for a set of interventions- a plausible coverage (defined as 60% of maximal achievable) and an aspirational coverage (defined as 95% of maximal achievable coverage). These scenarios were based on a consultation process during the development of the BMGF study and were designed to assess the sensitivity of the model and use case application to varying coverage values. The model can be applied to other populations or sub-populations though different choices of input parameters. These scenarios demonstrate model capability rather than constitute a validated policy recommendation.

#### Setting.

We ran the model for LMICs, grouped into three aggregated settings: Sub-Saharan Africa, South Asia and other LMICs. The parameters associated with each modelled setting represent the aggregate of LMICs that it contains. These were chosen to align with a previous BMGF study [13].

#### Interventions.

Interventions included in this analysis were based on the recent BMGF study. Six existing interventions and three emerging interventions (i.e., likely to be available in the near term) were included in the modelling analysis, described in detail in Table 2. Evidence for the impact of these interventions is given in Table 2.

As the novel interventions have not yet been widely scaled up, there is some uncertainty regarding their effect sizes. For the purposes of this study, effectiveness parameters for these interventions were informed by a combination of expert advice and values drawn from published literature. The effect size for the non-invasive haemoglobin (Hb) test is based on a theoretical maximum sensitivity, assuming that 100% sensitivity is not attainable in practice (representing a best-case scenario for such a test). The effect size for pre-eclampsia risk screening and prevention is derived from published evidence [14], adjusted to account for screening sensitivity. For AI-assisted ultrasound, the assumed effect size is equivalent to that of standard-of-care (SOC) ultrasound; however, the model incorporates higher coverage to reflect the anticipated increase in accessibility associated with this technology.

#### Scenarios design.

MNCH outcomes were projected across LMICs from 2024-2040 under the following intervention scenarios:

- **Baseline:** Interventions remain at current levels
- **Baseline + individual intervention scale-up:** Individual products scaled up in isolation to reach either 60% or 95% coverage by 2030 (except for SOC Ultrasound which was scaled up to 50%, see Table 2)
- **SOC scale up:** All SOC interventions scaled up together to reach either 60% or 95% by 2030.
- **SOC + novel diagnostics scale up:** All SOC plus novel diagnostics interventions scaled up together to reach either 60% or 95% by 2030.

In cases where the SOC product has baseline coverage greater than 60%, coverage was maintained for the 60% coverage scenarios.

#### Data.

Model inputs for demographic, epidemiological, and health system parameters were taken from available primary data sets, IHME GBD 2019 estimates, and published literature (Tables A, B and C in S1 Appendix). All models used region-specific data inputs where possible for three regional groupings: South Asia; sub-Saharan Africa; and other LMICs comprising countries in Latin America, North Africa/Middle East, and East/Southeast Asia/Oceania. We based intervention effect size assumptions on published literature and available primary data. Coverage parameter values were constrained by intervention delivery channel access (e.g., antenatal care coverage, in-facility delivery coverage).

## Results

### Comparison between individual intervention scenarios

In the baseline scenario, there were an estimated 5.9 billion DALYs from the modelled conditions in LMICs over 2024–2030 (Table 3)**.** Indirect impacts made up the majority of DALYs averted (~93% cumulative in the 60% coverage scenario and ~91% cumulative in the 95% coverage scenario), except for RUTF scale-up which impacts only infants directly in the model. From the individual intervention scenarios, the AI ultrasound had the greatest impact, followed by the SOC ultrasound, due to their large downstream indirect impacts. The AI ultrasound performed better due to higher possible coverage, as it could be performed by lower cadre healthcare workers. For the 95% scenarios, the highest impact interventions in terms of DALY reductions were AI ultrasound, SOC ultrasound and the preeclampsia screening.

**Table 3:** Reduction of total DALYs in each scenario by region compared to the baseline scenario.

Scenario	Impact type	Total DALYs averted (compared to baseline)	
		**60% + individual intervention scale up (Plausible scenarios)**	**95% + individual intervention scale up (Aspirational scenarios)**
*Baseline*	*Total (not averted)*	*6,317,221,237*	*6,317,221,237*
Baseline + SOC RUTF	Direct	0	3,156,725
	Indirect	0	0
Baseline + SOC Iron tablets	Direct	1,185,059	1,798,971
	Indirect	7,990,134	11,598,604
Baseline + SOC Hb test	Direct	0	32,262
	Indirect	0	209,850
Baseline + SOC preeclampsia urine dipstick test	Direct	6,927	148,910
	Indirect	46,617	615,537
Baseline + SOC ultrasound	Direct	8,195,552	8,195,552
	Indirect	93,237,748	93,237,748
Baseline + SOC preeclampsia treatment	Direct	0	0
	Indirect	0	654,049
Baseline + non-invasive Hb test	Direct	168,956	192,349
	Indirect	1,123,270	1,284,958
Baseline + preeclampsia screening and treatment	Direct	1,182,011	1,182,011
	Indirect	58,248,758	58,248,758
Baseline + AI ultrasound	Direct	10,831,668	20,669,950
	Indirect	122,577,369	227,395,843
SOC Scale up	Direct + Indirect	110,578,189	121,423,390
SOC + diagnostics scale up	Direct + Indirect	207,180,195	365,294,286

In particular, in the 95% scenario, AI ultrasound reduced DALYs attributable to birth asphyxia by 12%, obstructed labour by 11% and hemorrhage by 8%; SOC ultrasound reduced DALYs attributable to birth asphyxia by 5%, obstructed labour by 5%, and hemorrhage by 3%; preeclampsia screening reduced DALYs attributable to preeclampsia by 5%, stillbirth by 3% and non-RDS preterm by 1%.

### Scale up scenarios

Compared to the baseline scenario, scaling up all SOC interventions to 60% or 95% coverage averted an additional 111 million and 121 million DALYs over 2024–2040, respectively (Table 3, Fig 2).

**Fig 2:**
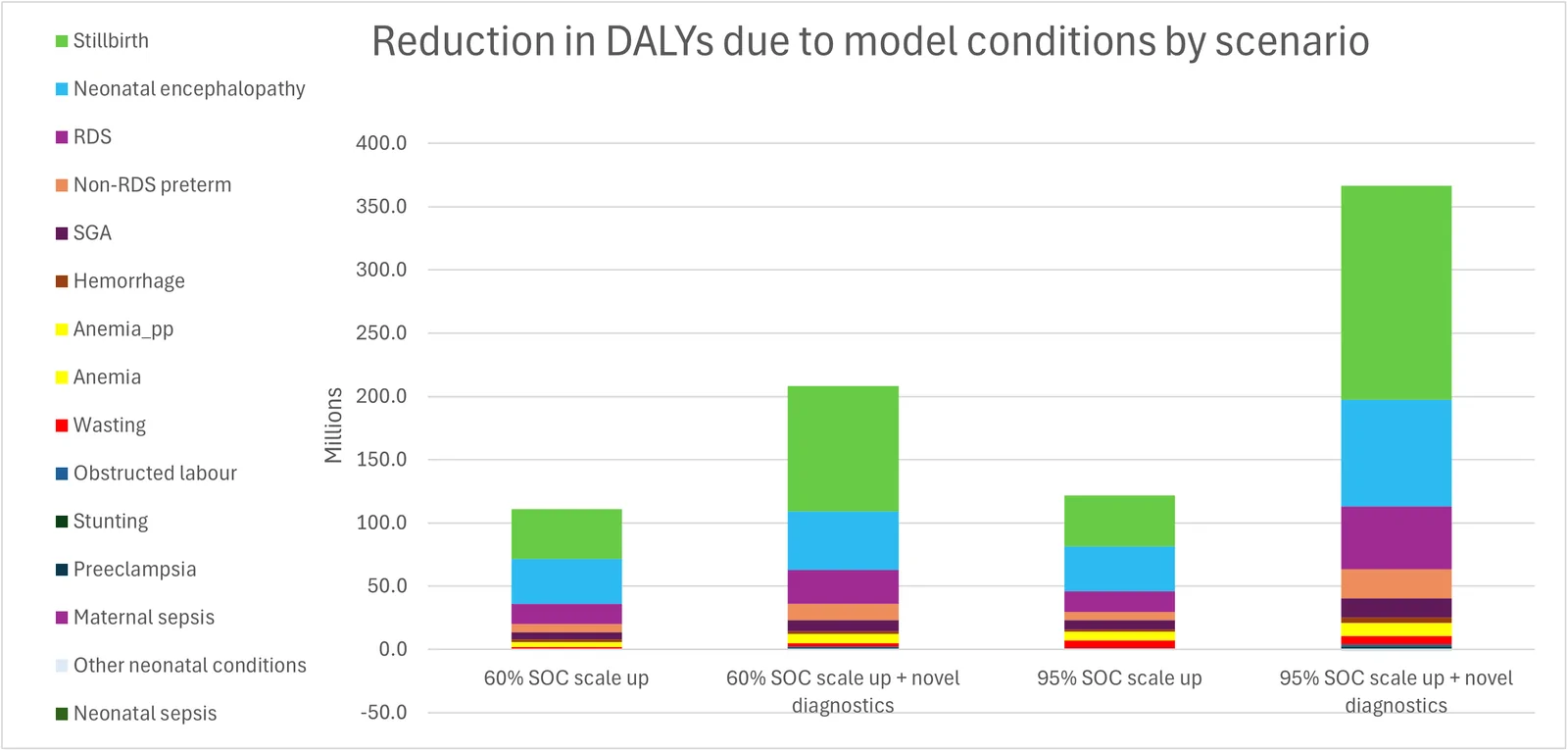
Reduction in DALYs by condition and scenario. Note: a negative value appears on the y-axis due to an increase of DALYs associated with infant conditions (wasting) relative to baseline, since the total number of pregnancies continuing to infancy is increased due to reductions in maternal and neonatal mortalities. Since no interventions directly target infant conditions, the total number of morbidities and mortalities (and therefore DALYs) associated with the conditions is increased.

Including novel diagnostic products in the scale up scenario had a considerable impact on mortalities and stillbirths averted compared to the baseline scenario (Fig 3). Scaling up all SOC and novel interventions to 60% or 95% coverage averted an additional 207 million and 365 million DALYs compared to the baseline, respectively (Table 3, Fig 2). In particular, including novel diagnostics at 60% coverage had a greater impact than the 95% coverage SOC scale up scenario.

**Fig 3:**
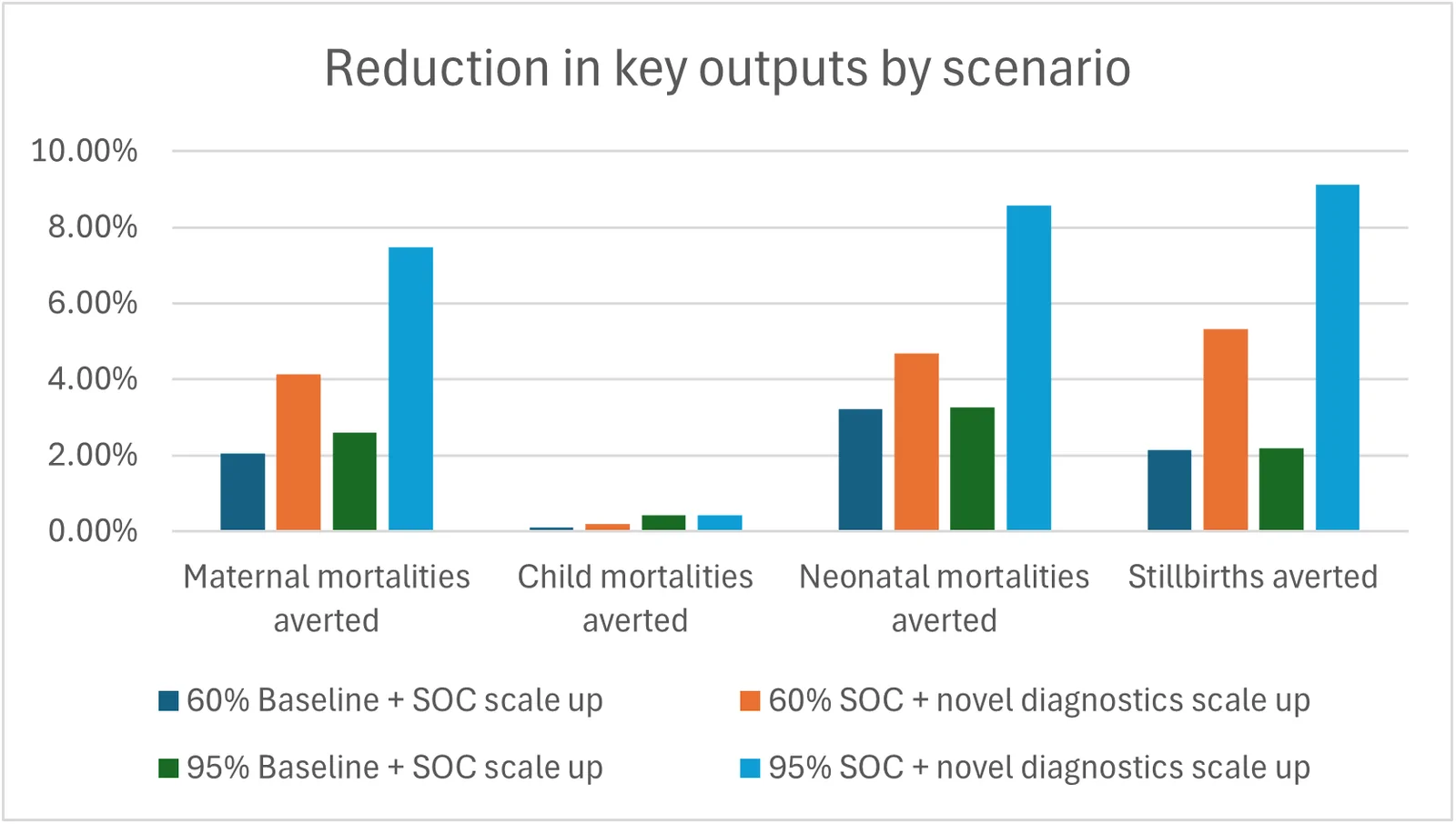
Reduction in key model outcomes for scale up scenario compared to baseline 2024-2040.

For the set of interventions considered in this example, the conditions with the greatest reduction in DALYs were stillbirths, wasting and birth asphyxia. The majority of the DALY reductions for these conditions were due to scaling up AI ultrasound, preeclampsia screening, SOC RUTF and SOC ultrasound.

It is worth noting that these scenarios resulted in a slight increase in infant DALYs as shown in Fig 2 (but fewer DALYs overall). This reflects a survivorship effect, whereby an increase in maternal and neonatal survival (and fewer neonatal death-related DALYs) lead to a greater number of newborns surviving into infancy, combined with the absence of interventions targeting infant conditions in this use case. However, the proportional incidence and case fatality rate are equal in all scenarios including the baseline.

### Reduction in mortalities/stillbirths by scenario

### Validation

There are very few other studies available to validate these outcomes against as the interventions have not been extensively studied. However, from those studies that we could identify, the impacts were relatively comparable. For example, Ward et al.[21] estimate that scaling up a comprehensive set of interventions, including family planning and community-based interventions, could avert approximately 197 thousand maternal deaths per annum globally by 2030. Similarly, multi-country LiST analyses [22] project that when a set of 28 interventions were scaled up in 27 LMICs over a 20-year time-period, over 50 million DALYs could be averted. Noting that these studies had different country scopes and time frames to our analysis, they are similar orders of magnitude in terms of DALYs averted to our estimate that globally over a 16-year time frame, 207 million DALYs could be averted from scaling up five interventions (including three high impact novel interventions). Our analysis also explicitly incorporates neonatal and child mortality, maternal and child morbidity, and prevention effects from emerging interventions, leading to additional DALYs captured.

## Discussion

We have created a model that captures dynamic interactions between MNCH conditions, including across trimesters, to account for the full impact of early detection and prevention of conditions during pregnancy. In addition, the model quantifies direct and indirect health benefits and allows for flexible integration of new interventions to enable the assessment of products under development.

A use case has been presented as an example. The example analysis shows there is potential to avert a large number of DALYs through greater coverage of existing evidence-based interventions. If the coverage of SOC interventions in LMICs could be increased to 60%, 111 million DALYs are estimated to be averted over 2024–2040 compared to the SOC scenario. This increased to 121 million DALYs averted if intervention coverage were increased to 95% by 2030, with diminishing returns due to ANC coverage restricting the widespread delivery of some interventions. Investing in increasing the coverage of SOC interventions is projected to have a significant impact on MNCH outcomes.

Additionally, the analysis shows that investment in novel diagnostic interventions is projected to substantially reduce DALYs in the model. The scenario with SOC and novel diagnostics scaled to 60% had a bigger impact on key outputs than scaling SOC interventions to an optimistic 95%. If both SOC interventions and novel diagnostic products were increased to 60% coverage in LMICs, 207 million DALYs could be averted over 2024–2040 compared to the baseline scenario. Scaling up the entire package of interventions to an aspirational 95% produced additional benefits. Of the novel interventions considered, AI ultrasound had the largest modeled impact, through the possibility of increased accessibility compared to the SOC ultrasound. When scaled up to 95% coverage in isolation, AI ultrasound averted 248 million DALYs in LMICs over 2024–2040 which was 68% of the DALYs averted in the SOC + novel diagnostics scale up with 95% coverage scenario. These results showed that even at low coverage levels investing in novel interventions were estimated to yield greater reductions in DALYs than focusing on increasing the coverage levels on current interventions. In order to make progress towards the SDG targets the model suggests that prioritising higher-yield interventions may maximise health gains. This analysis also highlights the importance of capturing downstream impacts, as the majority of benefits arose from indirect DALYs averted. By incorporating these effects, the model can be used to inform decision-making about interventions that consider their longer-term and broader health consequences.

An earlier version of this model was used to produce outcomes as part of a recent report for the BMGF which highlighted maternal and neonatal mortality worldwide [13]. The report notes that progress has stalled and offers insights into innovations needed to improve maternal, neonatal and childhood health and that reaching the SDG targets 3.1 and 3.2 will require significant improvement and investment in interventions with the highest impact. The report analysed how novel interventions might fast-track progress towards these targets. Modelling plausible intervention coverage rollout targets can identify where the largest impact and potential improvements can be, which can be used to support investment decisions. We hope that the model described in this paper will aid in decision making surrounding MNCH interventions and therefore help reduce the global burden of maternal, neonatal and child health conditions as policy makers aim to reach these targets.

The model used in this study has some similarities and differences to other models in the field. Some other tools that have been used to answer questions surrounding MNCH are the Lives Saved Tool (LiST) [3], Equitable Impact Sensitive Tool (EQUIST) [23], One Health Tool, Optima [24], Maternal and Newborn Directed Assessment of Technology (MANDATE) [25], Health Equity Assessment Toolkit (HEAT and HEAT Plus) [26] and REDUCE-ALIVE [24]. Depending on the country context and key questions of decision-makers, one tool may be more appropriate to use than another. In particular, LiST has been successfully used in a number of country studies to support funding bodies and policy makers in decisions surrounding the impact of WHO recommended interventions [27]. LiST is used to scale up key maternal and child health interventions in order to model health impacts and cost scenarios. The model presented in this paper shares a number of similarities with LiST. In both models, modifying coverages of interventions produces estimates of maternal and child (up to 5 years) deaths, mortality rates/ratios, deaths averted, intermediate outcomes, and single- and multiple-country scenarios. Intervention coverages can be projected into the future in order to model a number of different scenarios. However, they contrast in the way they calculate health outcomes in populations that are not directly targeted by a particular intervention. EQUIST, HEAT and HEAT-PLUS focus on including equity into policy and coverage decisions related to MNCH. Currently the model developed in this paper does not consider equity in its analysis. This is a possible direction for the future.

A unique strength of this model is the ability to flexibly implement new interventions. This allows a user to model the potential impact of novel interventions as well as a wide range of current interventions without requiring external developers to update the model. In cases where there is limited funding for research and development it is critical to understand the capacity for novel interventions and interventions that have not yet made it to market to impact the entire MNCH area. A further advantage of this model is that it includes trimester timesteps. This means that we can more accurately model early detection and treatment of conditions throughout pregnancy and the associated benefits. This can have further effects on downstream conditions in the maternal, neonatal and child populations. Additionally, as discussed, the model is set up to allow interactions between conditions. In such a way the complex interaction and downstream impacts of conditions may be used to test potential impact pathways. This may be advantageous to policy makers who are interested in the way conditions and packages of interventions interact.

As an example, AI ultrasound is a novel intervention which has a wider coverage than traditional ultrasound due to its ability to be used by a larger number of health care workers. Due to the model structure, we were able to integrate this novel intervention to better understand its potential impacts. Although AI ultrasound only targets pregnant women in the ANC module, it has the majority of its impacts downstream. This is due to treatment of conditions which are detected through AI ultrasound. Furthermore, trimester timesteps within the model allow for early AI ultrasound to detect and indicate early treatment for conditions during pregnancy, which may reduce adverse outcomes during childbirth, in neonates and in children under 5 years.

There are some limitations to this study. Firstly, in the theoretical intervention coverage scenarios, implementation challenges were not considered, including the adequacy of healthcare workforces and infrastructure to scale up interventions. Secondly, analyses were performed on large aggregated LMIC regions and so may not capture the intricacies of health systems related to individual countries. Country-specific analyses would be warranted with a broader set of interventions to inform policy. Thirdly, this study does not include consideration of costs or cost-effectiveness, which could be the subject of further analyses. Fourthly, although it would be useful to validate the outcomes of this use case against a similar analysis in existing models such as LiST, this is not possible due to the consideration of novel interventions. Similarly, we cannot properly identify the extent to which indirect effects of interventions are underestimated, since in reality we cannot easily identify which impacts are due to upstream impacts. Some future work may involve modelling the extent to which these indirect effects are under calculated. Additional future work may involve using the underlying Atomica software package to produce allocative efficiency analyses in-order-to aid decisions surrounding which interventions to prioritise. Fourthly, the current analysis does not explicitly incorporate equity considerations, such as access to healthcare, socioeconomic disparities, or variation in health gains across population subgroups. Future studies should both expand the range of downstream impacts included and integrate equity-relevant considerations to better capture the benefits arising from investment MNCH interventions. Finally, effect size assumptions for some novel interventions were informed in part by expert judgement, given the limited evidence base for products not yet widely deployed. Future analyses using this model may require more robust inputs for effect sizes.

## Conclusion

We have presented a mathematical modeling tool which can project the direct and indirect impact of packages of existing and novel interventions on a range of MNCH outcomes. The model can be flexibly adapted to include new interventions, and can be applied at a global, regional, national or sub-national level. Our example analysis identified that there is significant opportunity to avert morbidities, mortalities and DALYs in LMICs in modelled scenarios by expanding access to SOC and novel MNCH interventions.

## Supporting information

S1 Appendix Contains detailed information on the data inputs for the model and analysis and the model design. (DOCX)

S1 Checklist ISPOR checklist. (DOCX)

S1 Table Model outputs for the 60% coverage scenario. (XLSX)

S2 Table Model outputs for the 95% coverage scenario. (XLSX)
