# Supplementary material for: A coupled model to value indirect impacts of maternal, newborn and child health interventions
Source: PLOS Glob Public Health. 2026 Nov 6;6(11):e0005507. doi: 10.1371/journal.pgph.0005507 (PMC13742954; doi:10.1371/journal.pgph.0005507)
Supplement: Supplementary file 1 [file pgph.0005507.s001.docx]

# Supplementary material

A. Detailed model inputs

### Calibration data

*Table A. Data used for the calibration of the model*

|  | Value | Source |
| --- | --- | --- |
| Childbirth data |  |  |
| Pregnancies (2019 values) | SSA = 41,456,019  SA = 36,366,127  Other LMICs = 56,174,316 | GBD 2017(1) (live births inflated by 10%, since pregnancy data is a point prevalence) |
| Live Births | SSA = 37,687,290  SA = 33,060,115  Other LMICs = 51,067,560 | GBD 2017(1) |
| Stillbirths | SSA = 690,562  SA =544,298  Other LMICs =341,665 | GBD 2017(2) |
| Maternal data |  |  |
| Maternal hemorrhage cases | SSA = 5,365,475  SA = 3,113,735  Other LMICs = 4,869,693 | GBD 2017(2) |
| Maternal hemorrhage mortalities | SSA = 16,921  SA = 21,507  Other LMICs = 7,788 | GBD 2017(3) |
| Maternal obstructed labor and uterine rupture cases | SSA = 1,698,352  SA = 2,530,142  Other LMICs = 3,141,355 | GBD 2017(2) |
| Maternal obstructed labor and uterine rupture mortalities | SSA = 5,646  SA = 3,588  Other LMICs = 1,268 | GBD 2017(3) |
| Maternal sepsis and other maternal infections cases | SSA = 5,097,503  SA = 5,495,269  Other LMICs = 7,287,682 | GBD 2017(2) |
| Maternal sepsis and other maternal infections mortalities | SSA = 11,592  SA = 2,798  Other LMICs = 2,346 | GBD 2017(3) |
| Preeclampsia cases | SSA = 8,163,672  SA = 3,838,487  Other LMICs = 4,421,601 | GBD 2017(2) |
| Preeclampsia mortalities | SSA = 10,872  SA = 10,338  Other LMICs = 6,317 | GBD 2017(3) |
| Neonatal and infant data |  |  |
| Preterm cases | SSA = 4,529,977  SA = 4,684,608  Other LMICs = 4,657,171 | GBD 2017(2) (Neonatal preterm cases) |
| Neonatal preterm mortalities | SSA = 247,970  SA = 233,422  Other LMICs = 160,641 | GBD 2017(3) (Neonatal preterm cases) |
| Preterm non-respiratory distress syndrome mortalities | SSA = 86,790  SA = 81,698  Other LMICs = 56,224 | Assumption of 35% of preterm mortalities are non-RDS related |
| Preterm respiratory distress syndrome mortalities | SSA = 161,181  SA = 151,725  Other LMICs = 104,417 | Assumption of 65% of preterm mortalities are RDS related |
| Birth asphyxia cases | SSA = 804,020  SA = 316,905  Other LMICs = 192,010 | GBD 2017(2) (Neonatal encephalopathy due to birth asphyxia and trauma cases) |
| Neonatal encephalopathy mortalities due to birth asphyxia | SSA = 281,278  SA = 204,537  Other = 69,463 | GBD 2017(3) (Neonatal encephalopathy due to birth asphyxia and trauma mortality) |
| Neonatal sepsis cases | SSA = 1,732,108  SA = 1,428,840  Other LMICs = 2,807,923 | GBD 2017(2) (Neonatal sepsis and other neonatal infections cases) |
| Neonatal sepsis mortality | SSA = 122,962  SA = 55,814  Other LMICs = 43,539 | GBD 2017(3) (Neonatal sepsis and other neonatal infections mortality) |
| Wasting DALYs | SSA = 27,357,090  SA = 11,065,216  Other LMICs = 6,842,279 | GBD 2019(4) |
| Stunting DALYs | SSA = 3,520,709  SA = 1,620,418  Other LMICs = 903,219 | GBD 2019(4). Total DALYs attributable to stunting by region in 2019 among children under 1 year old |
|  |  |  |

### Model parameters

*Table B. Forecasted annual number of pregnancies*

|  | **2020** | **2021** | **2022** | **2023** | | **2024** | **2025** | **2026** | **2027** | **2028** | **2029** |
| --- | --- | --- | --- | --- | --- | --- | --- | --- | --- | --- | --- |
| **SA** | 55,258,931 | 54,630,123 | 54,009,212 | 53,360,556 | | 52,643,520 | 51,907,310 | 51,186,210 | 50,542,151 | 49,950,174 | 49,376,693 |
| **SSA** | 42,257,609 | 43,014,448 | 43,733,763 | 44,411,002 | | 45,024,866 | 45,592,479 | 46,118,691 | 46,602,469 | 47,047,692 | 47,444,945 |
| **Other** | 55,258,931 | 54,630,123 | 54,009,212 | 53,360,556 | | 52,643,520 | 51,907,310 | 51,186,210 | 50,542,151 | 49,950,174 | 49,376,693 |
|  | **2030** | **2031** | **2032** | **2033** | | **2034** | **2035** | **2036** | **2037** | **2038** | **2039** |
| **SA** | 48,836,726 | 48,333,763 | 47,894,049 | 47,498,299 | | 47,144,095 | 46,835,644 | 46,574,769 | 46,387,874 | 46,152,311 | 45,932,299 |
| **SSA** | 47,797,476 | 48,101,978 | 48,372,827 | 48,611,481 | | 48,813,495 | 48,980,365 | 49,109,069 | 49,220,958 | 49,313,024 | 49,383,894 |
| **Other** | 48,836,726 | 48,333,763 | 47,894,049 | 47,498,299 | | 47,144,095 | 46,835,644 | 46,574,769 | 46,387,874 | 46,152,311 | 45,932,299 |
|  | **2040** | **2041** | **2042** | **2043** | | **2044** | **2045** | **2046** | **2047** | **2048** | **2049** |
| **SA** | 45,720,951 | 45,513,709 | 45,313,056 | 45,053,556 | | 44,780,211 | 44,475,057 | 44,126,631 | 43,715,389 | 43,333,232 | 42,942,139 |
| **SSA** | 49,439,765 | 49,481,043 | 49,510,256 | 49,516,680 | | 49,499,257 | 49,470,617 | 49,433,728 | 49,387,982 | 49,323,324 | 49,236,703 |
| **Other** | 45,720,951 | 45,513,709 | 45,313,056 | 45,053,556 | | 44,780,211 | 44,475,057 | 44,126,631 | 43,715,389 | 43,333,232 | 42,942,139 |
|  | **2050** | **Source** | | |  |  |  |  |  |  |  |
| **SA** | 42,529,567 | GBD 2017(1) (live births inflated by 10%, since pregnancy data is a point prevalence) | | |  |  |  |  |  |  |  |
| **SSA** | 49,139,377 |  |  |  |  |  |  |  |  |  |  |
| **Other** | 42,529,567 |  |  |  |  |  |  |  |  |  |  |

*Table C. Data and sources used as inputs for the model*

|  | **Value** | **Provenance** |
| --- | --- | --- |
| Antenatal care |  |  |
| Number of pregnancies that occur annually |  | Calculated using live births |
| Proportion of births with mortality due to all childbirth conditions not captured in any module | SA = 0.0048  SSA = 0.0670  Other = 0.0700 | Calibrated to live births |
| Life expectancy | 88.9 | GBD 2019(5) |
| Average maternal age | 26 | Fall et al. Lancet Glob Health 2015.(6) |
| Proportion that receive antenatal care at any point during pregnancy | SA = 95%  SSA = 92%  Other = 98% | GBD 2019(7) |
| Proportion of pregnancy loss for any reason 1st trimester | SA = 0.035  SSA = 0.01  Other = 0.01 | Ahmed, Imran et al. The Lancet Global Health. 2018.(8) |
| Proportion of pregnancy loss for any reason 2nd trimester | SA = 0.01  SSA = 0.006  Other = 0.005 | Ahmed, Imran et al. The Lancet Global Health. 2018.(8) |
| Proportion of pregnancy loss for any reason 3rd trimester | SA = 0.033  SSA = 0.018  Other = 0.01 | Ahmed, Imran et al. The Lancet Global Health. 2018.(8) |
| Proportion of births at a referral facility not a result of risks detected via ultrasound (ie, those that have childbirth at a referral facility without a referral) | 0 | Assumption |
| Anemia |  |  |
| Anemia |  |  |
| Proportion of pregnant women attending ANC who received an ultrasound by 3rd trimester | 0.234 | Custom analysis on primary data from ARC PRiSMA.(9) |
| Relative risk of bleeding related anemia from hemorrhage | 10 | Ehwerhemuepha et al. Data in brief. 2022.(10) |
| Relative risk of bleeding related anemia from moderate or severe anemia during pregnancy | 5 | Ehwerhemuepha et al. Data in brief. 2022.(10) |
| Relative risk of bleeding related anemia from mild anemia during pregnancy | 2.5 | Ehwerhemuepha et al. Data in brief. 2022.(10) |
| Proportion of pregnant women that will experience anemia (total) | SA = 76.30%  SSA = 38.20%  Other = 28.00% | Custom analysis of the PRISMA pregnancy cohort data available in 2023 (second trimester proportions used as proxy)(9) |
| Proportion of anemia cases in pregnant women that will be mild in the absence of risk factors | 0.45 | GBD 2019(11) |
| Proportion of anemia cases in pregnant women that will be moderate | 0.53 | GBD 2019(11) |
| Proportion of anemia cases in pregnant women that will be severe | 0.025 | GBD 2019(11) |
| Proportion of pregnant women attending ANC in whom anemia will be diagnosed | 0.667 | SOC product efficacy |
| Proportion of mild anemia cases that are treated in the absence of intervention 2nd trimester | 0.05 | Assumption |
| Proportion of moderate anemia cases mitigated to mild anemia via treatment after 3 months in the absence of intervention 2nd trimester | 0.05 | Assumption |
| Proportion of severe anemia cases that are mitigated to moderate anemia via treatment after 3 months in the absence of intervention 2nd trimester | 0.05 | Assumption |
| Proportion with bleeding related anemia | 0.2 | Milman. Ann Hematol. 2011(12)  24% among non-supplemented women, rounded to 20% to include women anemic pre-partum |
| Disability due to mild anemia (YLDs per case) | 0.003 | GBD 2017(13) |
| Disability due to moderate anemia (YLDs per case) | 0.051 | GBD 2017(13) |
| Disability due to severe anemia (YLDs per case) | 0.147 | GBD 2017(13) |
| Hemorrhage |  |  |
| Relative risk of hemorrhage from non-severe anemia | 1.49 | BMGF Systematic Review and Meta-analysis |
| Relative risk of hemorrhage from severe anemia | 2.98 | BMGF Systematic Review and Meta-analysis |
| Relative risk of hemorrhage from obstructed labour | 2.44 | BMGF Systematic Review and Meta-analysis |
| Proportion of pregnant women with a risk factor for hemorrhage *identifiable* through ultrasound | 0.57 | Estimates from VITAL Pakistan Pregnancy Cohort. Ultrasound-identifiable risk factor for hemorrhage defined as presence of multiple, placenta previa, or breech presentation (breech is the only type of malpresentation measured in the database but this is the most common type)  Naz et al. BMJ Open 2023.(14) |
| Proportion of pregnant women with a risk factor for hemorrhage *identified* through ultrasound | 0.41 | Estimates from VITAL Pakistan Pregnancy Cohort. Ultrasound-identifiable risk factor for hemorrhage defined as presence of multiple, placenta previa, or breech presentation (breech is the only type of malpresentation measured in the database but this is the most common type)  Naz et al. BMJ Open 2023.(14) |
| Proportion accepting referral | 0.5 | Assumption |
| Relative risk of mortality due to hemorrhage not at a referral facility vs at a referral facility | 4.5 | Expert opinion |
| Proportion of childbirth in which hemorrhage will be imminent in the absence of risk factors | SA = 0.094  SSA = 0.131  Other = 0.089 | Calibrated to maternal hemorrhage cases |
| Proportion of childbirths with hemorrhage at a facility that result in maternal mortality in the absence of intervention | SA = 0.003  SSA = 0.003  Other = 0.001 | Calibrated to maternal hemorrhage mortalities |
| Disability weight due to hemorrhage at a referral facility (YLDs per case) | SA = 0.023  SSA = 0.0168  Other = 0.02 | GBD 2017(2, 13) (GBD estimates that 15% of maternal hemorrhage is severe and the disability weight is 0.324 while the disability weight for moderate is 0.114. Further, the duration for severe is 14 days and the duration for moderate is 7. Using the information on number of moderate/severe events and their associated disability, we estimate that half of maternal hemorrhage YLDs are due to each severity.  This is used estimate YLDs for moderate and YLDs for severe and divide by the number of incident episodes to get the reported values.) |
| Disability weight due to hemorrhage in other facility (YLDs per case) | SA = 0.132  SSA = 0.095  Other = 0.113 | As above |
| Maternal sepsis |  |  |
| Product risk reduction on maternal sepsis | 0.84 | Product effect size value |
| Post childbirth disability due to maternal sepsis (YLDs per case) | SA = 0.0047  SSA = 0.00418  Other = 0.004 | GBD 2017(2) (It is assumed that 95% of the YLDs per case are post-delivery since GBD does not include YLD breakdown by timing of sepsis.) |
| Obstructed labour |  |  |
| Proportion of pregnant women with risk factor for obstructed labor identified through ultrasound accepting referral | 0.75 | Custom analysis on primary data from ARC PRiSMA. VITAL pakistan pregnancy cohort shown for reference.  Naz et al. BMJ Open 2023.(14) |
| Proportion of pregnant women with a risk factor for obstructed labour *identifiable* through ultrasound | 0.9 | Custom analysis on primary data from ARC PRiSMA (Note only breech presentation was measured in the dataset, but this is the most common type of malpresentation.) VITAL pakistan pregnancy cohort shown for reference.  Naz et al. BMJ Open 2023.(14) |
| Relative risk of mortality due to obstructed labour not at a referral facility vs at a referral facility | 2 | Assumption |
| Proportion of obstructed labour births that require a c-section | 1 | Assumption |
| C-section coverage |  | GBD 2019. Estimates of C-section prevalence and In-facility delivery. |
| Proportion of childbirth in which obstructed labour will be imminent | SA = 0.076  SSA = 0.042  Other = 0.057 | Calibrated to maternal obstructed labor and uterine rupture cases |
| Proportion of births at a referral facility that result in mortality due to obstructed labor | SA = 0.076  SSA = 0.042  Other = 0.057 | Calibrated to maternal obstructed labor and uterine rupture mortalities |
| Less severe disability due to obstructed labour in referral facility (DALYs per case) | SA = 0.33  SSA = 0.056  Other = 0.021218 | GBD 2017(2) |
| Disability due to obstructed labour in other location (DALYs per case) | SA = 0.066  SSA = 0.112  Other = 0.042436 | Assumed to be double the disability weight of obstructed labour at a referral facility. |
| Preeclampsia |  |  |
| Proportion preeclampsia cases that are high risk | 0.1 | Assumption |
| Preeclampsia diagnostic tool sensitivity | 1 | Model assumption |
| Proportion of pregnancies with preeclampsia that are early onset | 0.3 | Magee et al. PLOS Med 2018.(15) |
| Relative risk of mortalty due to untreated preeclampsia 2^nd^ trimester vs untreated non-severe preeclampsia 3^rd^ trimester | 1 | Assumption |
| Relative risk of mortalty due to untreated severe preeclampsia 3^rd^ trimester vs untreated non-severe preeclampsia 3^rd^ trimester | 2 | Assumption |
| Proportion of cases of late onset preeclampsia that are severe | 0.05 | Abalos et al. The Lancet Global Health 2013.(16) |
| Preeclampsia product efficacy for non-severe pre-eclampsia 3rd trimester | 0.001 | Model assumption |
| Proportion of pregnancies in which preeclampsia will occur | SA = 0.110  SSA = 0.195  Other = 0.079 | Calibrated to pre-eclampsia cases |
| Mortality rate for untreated preeclampsia 2nd trimester | SA = 0.003  SSA = 0.001  Other = 0.001 | Calibrated to pre-eclampsia mortalities |
| Disability weight due to preeclampsia | 0.0092 | GBD 2017(17) |
| Asphyxia |  |  |
| Relative risk of mortality due to neonatal encephalopathy not at a referral facility vs at a referral facility | 2 | Assumption |
| Proportion of neonates with birth asphyxia | SA = 0.0147  SSA = 0.0305  Other = 0.0099 | Calibrated to neonatal encephalopathy due to birth asphyxia and trauma cases |
| Proportion of asphyxia at a referral facility that results in mortality due to neonatal encephalopathy | SA = 0.4099  SSA = 0.2199  Other = 0.3639 | Calibrated to neonatal encephalopathy due to birth asphyxia and trauma mortality |
| Less severe disability due to encephalopathy (treated) (YLDs per case) | 0.061 | GBD 2017(13) (moderate motor impairment due to neonatal encephalopathy) |
| Disability due to encephalopathy (YLDs per case) | SA = 2.17  SSA = 2.28  Other = 3.53 | GBD 2017(2) |
| Asphyxia |  |  |
| Relative risk of mortality due to neonatal encephalopathy not at a referral facility vs at a referral facility | 2 | Assumption |
| Proportion of neonates with birth asphyxia | SA = 0.0147  SSA = 0.0305  Other = 0.0099 | Calibrated to neonatal encephalopathy due to birth asphyxia and trauma cases |
| Proportion of asphyxia at a referral facility that results in mortality due to neonatal encephalopathy | SA = 0.4099  SSA = 0.2199  Other = 0.3639 | Calibrated to neonatal encephalopathy due to birth asphyxia and trauma mortality |
| Less severe disability due to encephalopathy (treated) (YLDs per case) | 0.061 | GBD 2017(13) (moderate motor impairment due to neonatal encephalopathy) |
| Disability due to encephalopathy (YLDs per case) | SA = 2.17  SSA = 2.28  Other = 3.53 | GBD 2017(2) |
| Neonatal sepsis |  |  |
| Relative risk of neonatal sepsis from maternal sepsis | 4.42 | Beck et al. Obstet Gynecol 2021.(18) |
| Proportion of preterm that is identified (and targeted for treatment to prevent neonatal sepsis) | 90.00% | Assumption |
| Relative risk of neonatal sepsis in neonates who are preterm or SGA versus term and AGA | 1 | Assumption |
| Proportion of childbirths in which neonatal sepsis occurs in term AGA neonates in the absence of treatment | SA = 0.044  SSA = 0.034  Other = 0.056 | Calibrated to maternal sepsis and other maternal infections cases |
| Neonatal sepsis mortality rate | SA = 0.040  SSA = 0.098  Other = 0.016 | Calibrated to maternal sepsis and other maternal infections moralities |
| Neonatal sepsis disability weight (DALYs per case) | SA = 0.634  SSA = 0.3  Other = 0.45 | GBD 2017(2) |
| Other neonatal conditions |  |  |
| Proportion of neonates with mortality due to other neonatal conditions | 0.002 | GBD 2017.(17) (Calculated as number of deaths under 28 days minus the number of deaths due to preterm, encephalopathy, and sepsis as a proportion of total number of live births globally.) |
| Preterm |  |  |
| Proportion accepting referral | 0.5 | Assumption |
| Preterm: Proportion of term births that are SGA | SA = 46%  SSA = 23%  Other = 9% | D.J. Erchick et al. BJOG 2023.(19) |
| Proportion of preterm births that are SGA | SA = 18%  SSA = 6%  Other = 10% | D.J. Erchick et al. BJOG 2023.(19) |
| Relative risk of SGA from anemia (moderate and severe) | 1.53 | Naoko et al. The Journal of Nutrition 2012.(20) |
| Relative risk of preterm from maternal early onset pre-eclampsia | 6.57 | [Abalos](https://obgyn.onlinelibrary.wiley.com/doi/epdf/10.1111/1471-0528.12629) et al. BJOG. 2014.(21) |
| Relative risk of preterm from maternal late onset pre-eclampsia | 2.44 | van Esch et al. The Journal of maternal-fetal and neonatal medicine 2017.(22) |
| Relative risk of SGA from maternal early onset pre-eclampsia | 2.1 | Bernardes et al. PLoS One 2020.(23) |
| Relative risk of SGA from maternal late onset pre-eclampsia | 1.1 | Bernardes et al. PLoS One 2020.(23) |
| Proportion of births that are preterm | SA = 0.040  SSA = 0.098  Other = 0.016 | Calibrated to neonatal preterm cases |
| Proportion of childbirths that will be preterm sga which are identified through ultrasound | 0.6 | Product effect size assumption |
| Proportion of childbirths that will be preterm aga which are identified through ultrasound | 0.5 | Product effect size assumption |
| Proportion of childbirths that will be term sga which are identified through ultrasound | 0.6 | Product effect size assumption |
| Proportion of childbirths that will be term aga which are identified through ultrasound | 0.5 | Product effect size assumption |
| Relative risk of mortality due to preterm not at a referral facility versus at a referral facility | 2 | Assumption |
| Relative risk of mortality due to preterm SGA vs term SGA | 3.85 | Hazel et al. BJOG 2023.(24) (Calculated as proportion with mortality due to preterm SGA/proportion with mortality due to term SGA) |
| Relative risk of mortality due to preterm AGA vs term SGA | 2.22 | Hazel et al. BJOG 2023.(24) (Calculated as proportion with mortality due to term SGA/proportion with mortality due to term SGA) |
| Proportion of cases of term SGA not at a referral facility that result in mortality | SA = 0.030  SSA = 0.025  Other = 0.018 | Calibrated to neonatal preterm mortalities |
| Disability due to preterm SGA (YLDs per incident case) | SA = 0.477  SSA = 0.407  Other = 0.72 | GBD 2017(2) |
| Disability due to preterm (YLDs per incident case) | SA = 0.477  SSA = 0.407  Other = 0.72 | GBD 2017(2) |
| Preterm: Disability due to SGA (YLDs per incident case) | SA = 0.477  SSA = 0.407  Other = 0.72 | GBD 2017(2) |
| RDS |  |  |
| Proportion of cases of preterm that are identified | 0.5 | Assumption based on expert opinion |
| Proportion of cases of respiratory distress syndrome in which the neonate is less than 28 weeks gestational age | 10% | Assumption based on expert opinion |
| Respiratory distress syndrome mortality rate for cases at a referral facility less than 28 weeks | 81% | Assumption based on expert opinion |
| Respiratory distress syndrome mortality rate for cases at other location less than 28 weeks | 100% | Assumption based on expert opinion |
| Respiratory distress syndrome mortality rate at referral in the absence of intervention greater than 28 weeks | 37.60% | Assumption based on expert opinion and analysis by evaluserve |
| Respiratory distress syndrome mortality rate for cases at other location greater than 28 weeks | 92.20% | Assumption based on expert opinion and analysis by evaluserve |
| Proportion of childbirth in which respiratory distress syndrome occurs | SA = 0.066  SSA = 0.064  Other = 0.049 | Calibrated to preterm respiratory distress syndrome mortalities |
| Disability weight due to respiratory distress syndrome morbidity | 0.175 | GBD 2017(13) |
| Stillbirth |  |  |
| Relative risk of stillbirth from obstructed labor | 1.87 | Custom analysis on primary data from CHAIN and KI wasting rally.  The Childhood Acute Illness and Nutrition (CHAIN) Network. The Lancet Global Health 2022.(25)  Mertens et al. BMJ 2021.(26) |
| Relative risk of stillbirth from maternal underweight | 1.1 | Custom analysis on primary data from CHAIN and KI wasting rally.  The Childhood Acute Illness and Nutrition (CHAIN) Network. The Lancet Global Health 2022.(25)  Mertens et al. BMJ 2021.(26) |
| Relative risk of stillbirth from maternal sepsis | 1.58 | Custom analysis on primary data from CHAIN and KI wasting rally.  The Childhood Acute Illness and Nutrition (CHAIN) Network. The Lancet Global Health 2022.(25)  Mertens et al. BMJ 2021.(26) |
| Relative risk of stillbirth from early onset pre-eclampsia | 35 | Harmon, et al. Obstet Gynecol 2015.(27) |
| Relative risk of stillbirth from late onset pre-eclampsia | 1.86 | van Esch et al. The Journal of maternal-fetal and neonatal medicine 2017.(22) |
| Proportion of births that are stillbirth in the absence of risk factors | SA = 0.0166  SSA = 0.0182  Other = 0.0067 | Calibrated to number of stillbirths |
| Stunting |  |  |
| Relative risk of stunting from preterm | 2 | Christian et al. IJE 2013(28) |
| Relative risk of stunting from SGA | 2 | Christian et al. IJE 2013(28) |
| Proportion of childbirths that result in stunting | SA = 0.577  SSA = 1  Other = 0.204 | Calibrated to stunting DALYs |
| Disability due to stunting (lifetime YLDs per infant) | 0.088 | GBD 2017(17) |
| Wasting |  |  |
| Proportion of births that are underweight 8 days to 5 months in the absence of risk factors | SA = 33%  SSA = 13%  Other = 10% | Custom analysis on primary data from KI wasting rally.  Mertens et al. BMJ 2021.(26) |
| Proportion of births that result in prolonged wasting 8 days to 5 months without intervention | 1 | Assumption |
| Relative risk of severe wasting 6 to 18 months vs moderate wasting 6 to 18 months | SA = 0.33  SSA = 0.35  Other = 0.32 | Custom analysis on primary data from KI wasting rally.  Mertens et al. BMJ 2021.(26) |
| Relative risk of moderate wasting 19 to 59 months vs moderate wasting 6 to 18 months | SA = 0.22  SSA = 0.25  Other = 0.06 | Custom analysis on primary data from KI wasting rally.  Mertens et al. BMJ 2021.(26) |
| Relative risk of severe wasting 19 to 59 months vs moderate wasting 6 to 18 months | SA = 0.83  SSA = 0.69  Other = 0.25 | Custom analysis on primary data from KI wasting rally.  Mertens et al. BMJ 2021.(26) |
| Proportion of births with moderate wasting 6 to 18 months from birth in the absence of risk factors | SA = 0  SSA = 0.056  Other = 0.003 | Calibrated to Wasting DALYs |
| Relative risk of wasting from SGA | 2.36 | Christian et al. IJE 2013(28) |
| Relative risk of wasting from preterm | 1.55 | Christian et al. IJE 2013(28) |
| Relative risk of wasting from preterm SGA | 2.36 | Assumed the highest of the preterm and SGA |
| Proportion of cases of wasting that result in mortality due to wasting 8 days to 5 months without intervention | 0.05 | Custom analysis on primary data from CHAIN and KI wasting rally.  The Childhood Acute Illness and Nutrition (CHAIN) Network. The Lancet Global Health 2022.(25)  Mertens et al. BMJ 2021.(26) |
| Proportion of births with severe wasting that result in hospitalized wasting 6 to 18 months from childbirth | 0.05 | Custom analysis on primary data from MAL-ED.  The MAL-ED Network Investigators. Clinical Infectious Diseases 2014.(29) |
| Proportion of births with moderate wasting that result in hospitalized wasting 6 to 18 months from childbirth | 0.015 | Custom analysis on primary data from MAL-ED.  The MAL-ED Network Investigators. Clinical Infectious Diseases 2014.(29) |
| Proportion of cases of hospitalized severe wasting that result in mortality 6 to 18 months from childbirth in the absence of intervention | 0.21 | Custom analysis on primary data from MAL-ED.  The MAL-ED Network Investigators. Clinical Infectious Diseases 2014.(29) |
| Proportion of cases of hospitalized moderate wasting that result in mortality 6 to 18 months from childbirth in the absence of intervention | 0.08 | Custom analysis on primary data from MAL-ED.  The MAL-ED Network Investigators. Clinical Infectious Diseases 2014.(29). |
| Proportion of cases of no illness/community based severe wasting that result in mortality 6 to 18 months from childbirth in the absence of intervention | 0.01 | Custom analysis on primary data from KI wasting rally.  Mertens et al. BMJ 2021.(26) |
| Proportion of cases of no illness/community based moderate wasting that result in mortality 6 to 18 months from childbirth in the absence of intervention | 0.01 | Custom analysis on primary data from KI wasting rally.  Mertens et al. BMJ 2021.(26) |
| Proportion of births with severe wasting that result in hospitalized wasting 19 to 59 months from childbirth | 0.05 | Assume same proportions as 6 to 18 months |
| Proportion of births with moderate wasting that result in hospitalized wasting 19 to 59 months from childbirth | 0.015 | Assume same proportions as 6 to 18 months |
| Proportion of cases of hospitalized severe wasting that result in mortality 19 to 59 months from childbirth in the absence of intervention | 0.21 | Assume same proportions as 6 to 18 months |
| Proportion of cases of hospitalized moderate wasting that result in mortality 19 to 59 months from childbirth in the absence of intervention | 0.08 | Assume same proportions as 6 to 18 months |
| Proportion of cases of no illness/community based severe wasting that result in mortality 19 to 59 months from childbirth in the absence of intervention | 0.01 | Assume same proportions as 6 to 18 months |
| Disability due to underweight (lifetime YLDs per infant due to underweight 8d – 5 months) | 0.0133 | GBD 2017(13) |
| Disability due to underweight (lifetime YLDs per infant due to wasting 6 – 18 months) | 0.033 | GBD 2017(13) |
| Disability due to underweight (lifetime YLDs per infant due to wasting 19 – 59 months) | 0.052 | GBD 2017(13) |

B. Model calibration

### Calibration methodology

This iteration of the model was calibrated to match specific outputs for mortalities, morbidities and DALYs as described in Table A. The values which the model is calibrated to are shown in Table A. These calibrated values were held constant in future projections.

### Model outputs

*Table D. 2019 Values produced by the model for key outputs with the percentage difference from calibratable data as described in Table A.*

|  | SSA | % difference | SA | % difference | Other LMICs | % difference |
| --- | --- | --- | --- | --- | --- | --- |
| **Maternal outputs** | | | | | | |
| Hemorrhage cases | 5,365,475 | 0.00% | 3,113,735 | 0.39% | 4,869,693 | 0.00% |
| Hemorrhage mortality | 16,914 | 0.00% | 21,506 | 0.01% | 7,788 | 0.00% |
| Obstructed labor cases | 1,698,352 | 2.86% | 2,530,142 | 1.10% | 3,141,355 | 3.55% |
| Obstructed labor mortality | 5,644 | 0.00% | 3,588 | 0.00% | 1,268 | 0.00% |
| Maternal sepsis cases | 5,097,503 | 0.00% | 5,461,385 | 0.00% | 7,287,682 | 0.00% |
| Maternal sepsis mortality | 11,587 | 0.04% | 2,798 | 0.00% | 2,346 | 0.00% |
| Preeclampsia cases | 8,163,672 | 0.00% | 3,838,487 | 0.00% | 4,421,601 | 0.00% |
| Preeclampsia mortality | 10,855 | 0.04% | 10,341 | 0.00% | 6,267 | 0.00% |
| **Maternal outputs** | | | | | | |
| Live Births | 37,930,415 | 0.00% | 32,801,183 | 0.62% | 50,859,144 | 0.00% |
| Stillbirths | 690,562 | 0.04% | 544,298 | 0.00% | 341,665 | 0.00% |
| **Neonatal and infant outputs** | | | | | | |
| Neonatal preterm birth | 4,404,048 | 0.64% | 4,736,873 | 0.79% | 4,828,542 | 0.41% |
| Preterm non-Respiratory distress syndrome mortalities | 86,791 | 0.00% | 81,698 | 0.00% | 56,225 | 0.00% |
| Preterm Respiratory distress syndrome mortalities | 161,188 | 0.00% | 151,505 | 0.14% | 105,942 | 1.44% |
| Birth asphyxia cases | 804,020 | 0.00% | 316,905 | 0.00% | 192,010 | 0.00% |
| Neonatal encephalopathy due to birth asphyxia mortality | 281,125 | 0.05% | 204,241 | 0.14% | 69,441 | 0.03% |
| Neonatal sepsis cases | 1,732,108 | 0.00% | 1,428,840 | 0.00% | 2,807,923 | 0.00% |
| Neonatal sepsis mortality | 122,846 | 0.09% | 55,733 | 0.14% | 43,270 | 0.62% |
| Wasting DALYs | 27,144,462 | 1% | 14,984,718 | 27% | 21,919,539 | 70% |
| Stunting DALYs | 3,164,688 | 11% | 1,569,022 | 6% | 895,895 | 8% |
| Proportion of births with wasting 8 days to 5 months | 13% | 0.00% | 33% | 0.00% | 10% | 0.00% |
| Proportion of preterm births that are SGA | 7% | 0.00% | 18% | 0.00% | 11% | 0.00% |
| Proportion of term births that are SGA | 16% | 0.16% | 51% | 0.03% | 8% | 0.79% |

Wasting calibration

Table C shows that the model outputs represent a good fit to most indicators with the exception of wasting. Wasting incidence was calibrated using two values. These were the wasting incidence in the 8 days to 5 months time period and the total wasting DALYs. There is uncertainty and a lack of data surrounding what proportion of wasting is occurring in the 8 days to 5 months time period. In the cases of South Asia and Other LMICs this resulted in more DALYs due to wasting in the 8 days to 5 month time period than were reported for children <5 years. Future work may address this by explicitly disaggregating wasting into different time periods.

C. Interventions

### Coverage inputs by scenario

*Table E. The maximal attainable coverage levels for each intervention under each scenario.* *Note that in the ‘+diagnostics’ scenario, the Hb test and AI ultrasound interventions displace their corresponding standard-of-care interventions, with affected coverages marked with an asterisk. Baseline coverages for the SOC interventions are based on expert opinion.*

|  | Coverage | | | | | | |
| --- | --- | --- | --- | --- | --- | --- | --- |
| Intervention | SOC (SSA) | SOC (SA) | SOC (Other) | SOC scale-up 60% | SOC + diagnostics scale up 60% | SOC scale-up 95% | SOC + diagnostics scale up 95% |
| SOC RUTF | 90% | 90% | 90% | 90% | 90% | 95% | 95% |
| SOC Iron tablets | 20% | 20% | 20% | 60% | 60% | 95% | 95% |
| SOC Pre-eclampsia urine dipstick test | 50% | 50% | 50% | 60% | 60% | 95% | 95% |
| SOC Ultrasound | 20% | 20% | 20% | 60% | 60% | 95% | *95% |
| SOC Pre-eclampsia treatment (severe PE only) | 60% | 60% | 60% | 60% | 60% | 95% | 95% |
| SOC Hb test | 40% (1^st^ trimester)  75% (2^nd^ trimester) | 50% (1^st^ trimester)  80% (2^nd^ trimester) | 65% (1^st^ trimester)  85% (2^nd^ trimester) | SA/SSA; 60%  Other; 65% |  | 95% | *95% |
| Non-invasive Hb test | - | - | - | - | 60% | - | 95% |
| Pre-eclampsia risk screening and prevention | - | - | - | - | 60% | - | 95% |
| AI ultrasound | - | - | - | - | 60% | - | 95% |

### Direct vs indirect impacts

Table F. Mapping between interventions and impacts on each condition, showing direct impacts (green) and indirect impacts (yellow).

|  | SOC RUTF | SOC Iron tablets | SOC Pre-eclampsia urine dipstick test | SOC Ultrasound | SOC Pre-eclampsia treatment | SOC Hb test | Non-invasive Hb test | Pre-eclampsia risk screening and prevention | AI ultrasound |
| --- | --- | --- | --- | --- | --- | --- | --- | --- | --- |
| Anemia |  |  |  |  |  |  |  |  |  |
| Postpartum anemia |  |  |  |  |  |  |  |  |  |
| Hemorrhage |  |  |  |  |  |  |  |  |  |
| Maternal sepsis |  |  |  |  |  |  |  |  |  |
| Birth asphyxia |  |  |  |  |  |  |  |  |  |
| Neonatal sepsis |  |  |  |  |  |  |  |  |  |
| Non- RDS preterm |  |  |  |  |  |  |  |  |  |
| Obstructed labour |  |  |  |  |  |  |  |  |  |
| Other neonatal conditions |  |  |  |  |  |  |  |  |  |
| Preeclampsia |  |  |  |  |  |  |  |  |  |
| RDS |  |  |  |  |  |  |  |  |  |
| SGA |  |  |  |  |  |  |  |  |  |
| Stilllbirth |  |  |  |  |  |  |  |  |  |
| Stunting |  |  |  |  |  |  |  |  |  |
| Wasting |  |  |  |  |  |  |  |  |  |

Table E. Direct (green) and indirect (yellow) impacts of individual products on outcomes within the model.

D. Detailed model description

Modelling was done using the Atomica compartment modelling toolbox, which is a simulation engine developed to flexibly implement compartment-based models of disease, and to analyze the effect of fundable interventions.

### Cause-specific modules

Modules are split into 4 sub-categories based on the timestep in which they occur as shown in Table G.

*Table G. Sub-categories by timing of modules within the model*

| **Time period** | Pregnancy trimesters | Childbirth | Neonatal time period | Infant time period up to 5-year |
| --- | --- | --- | --- | --- |
| **Collections** | Provision of maternal care throughout pregnancy &  Management of underlying risk factors during pregnancy | Maternal outcomes, Preterm & SGA | Neonatal outcomes | Infant outcomes |
| **Modules** | Antenatal care,  Anemia,  Pre-eclampsia | Obstructed labour, Hemorrhage,  Maternal sepsis & Preterm & SGA | RDS, Neonatal sepsis, Birth asphyxia (including encephalopathy), Stunting, Other neonatal conditions, Wasting (neonate) | Wasting (infant) |

The modules receive demographic and epidemiological data inputs from modules which are in an earlier timestep and within the same timestep allowing for interventions to have downstream impacts.

*Fig A. Overview of the model with connections between the modules.*

#### Population level interactions

An important feature of this model is its ability to incorporate interactions between populations. This allows condition and coverage level impacts to be carried from one population to another. If the prevalence of a condition is reduced by an intervention this has flow on effects for other conditions within the same population group. The population level interactions in the model were:

- Antenatal care to pregnancy modules
- Referral facility delivery to pregnancy modules
- Number of pregnancies to pregnancy modules
- Number of surviving pregnant women to childbirth modules
- Number of live births to neonatal modules
- Number of surviving neonates to infant modules

#### ANC module

*Fig B. Structure of the antenatal care module.*

*Overview:* The ANC module is a 12-month/4-timestep module that occurs during the pregnancy and in the 3-months postpartum time period and considers the population of pregnant women. For each trimester, women are classified as pregnant or no longer pregnant. Women who are no longer pregnant are removed from the cascade. In the first trimester pregnant women are split into ‘Ever attends ANC’ and ‘No ANC’ which are used as inputs in other modules which require ANC attendance for intervention delivery. Input data is ‘Attends ANC at any point during pregnancy.’ In the childbirth timestep, pregnant women are split into those that have a birth at a referral facility and those that do not have a birth at a referral facility.

*Interventions:* Outcomes can be influenced by the Ultrasound intervention, which determines how many pregnant women have an ultrasound. This targets pregnant women attending ANC.

*Outputs:* Pregnant women at the end of 3^rd^ trimester, ANC attendance and Birth at a referral facility for reasons other than identification of condition.

*Connection to other modules*: Outputs for ‘attends ANC’ connect to all pregnancy modules and impacts the proportion who attend ANC and outputs ‘referral facility delivery’ connects to all childbirth, RDS and asphyxia modules and impacts the proportion of births with a referral facility birth.

*Assumptions:* We make the following assumptions in this module:

- Attending ANC in the first trimester is an approximation for “ever attends ANC”.
- There is a low rate of loss-to-follow up for ANC attendance in first trimester continuing into 2^nd^ and 3^rd^ trimesters
- For annual pregnancies we use live births inflated by 10% (since the pregnancy data is a point prevalence rather than yearly)
- Timing of ultrasound has no impact on detection of a condition

#### Anemia module

*Fig C. Structure of the anemia module.*

*Overview:* The anemia module is a 12-month/4-timestep module that spans pregnancy and a 3-month postpartum time period, and captures the impact of anemia on pregnant women. For each trimester, women are classified as not anemic, or as having mild, moderate or severe anemia. DALYs are accrued at each trimester depending on severity. Women who have anemia in one trimester can move to a less severe category of anemia through treatment. Anemia status in the third trimester impacts post-partum outcomes, which include disability due to mild, moderate or severe anemia. If post-partum screening occurs, women with moderate or severe anemia post-partum can receive treatment which reduces the severity of post-partum anemia-related disability by one category. No deaths are directly attributable to anemia – however, anemia status has an impact on the hemorrhage module and indirectly increases the number of deaths due to hemorrhage.

*Outputs:* Total number of mild, moderate and severe anemia cases (third trimester), total number of post-partum anemia cases, total number of anemia DALYs.

*Interventions:* Outcomes can be influenced by diagnostic interventions (SOC Hb test and Non-invasive Hb test interventions) which increase the number of people eligible for treatment and thereby reduce the anemia severity. These both target pregnant women who attend ANC.

*Connection to other modules*: The prevalence of anemia impacts hemorrhage incidence (hemorrhage module), and the likelihood of preterm birth and/or SGA (preterm/SGA module).

*Assumptions:* We make the following assumptions in this module:

- Without interventions, anemia status is assumed to not change during the pregnancy
- New cases of post-partum anemia (e.g., caused by hemorrhage during childbirth) are assumed to be severe in the absence of post-partum treatment.
- Interventions reduce anemia by one severity level
- The probability of hemorrhage during childbirth is dependent only on anemia status at the time of childbirth.
- Diagnosis of anemia is independent of severity

#### Preeclampsia module

*Fig D. Structure of the preeclampsia module.*

*Overview:* The preeclampsia module is a 9-month/3-timestep module that occurs during the pregnancy and 3-months postpartum time period and considers the population of pregnant women. DALYs are accrued at each trimester with each outcome only included once. For the 1^st^ trimester, pregnant women are classified as either healthy with respect to preeclampsia, having imminent preeclampsia averted or with imminent preeclampsia. Pregnant women with preeclampsia successfully averted will not have preeclampsia during pregnancy and do not have a preeclampsia burden. Those that will have preeclampsia are classified as having either early onset preeclampsia (which occurs during the 2^nd^ trimester) or late onset preeclampsia which occurs during the 3^rd^ trimester. Those with late onset preeclampsia are further split by severity level. All cases can have disability averted via treatment. Untreated preeclampsia results in morbidity or mortality.

*Outputs:* Outputs are total number of healthy women with respect to preeclampsia, cases of preeclampsia averted via prevention treatment, cases of late onset preeclampsia which are treated or untreated, mortality due to 3^rd^ trimester preeclampsia (late onset), cases of 2^nd^ trimester (early onset) preeclampsia which are treated or untreated, mortality due to 2nd trimester preeclampsia (early onset), DALYs due to preeclampsia.

*Interventions:* Outcomes can be influenced by risk prediction and risk prevention, which target pregnant women in the 1^st^ trimester, as well as urine dipstick test and preeclampsia treatment, which target pregnant women in the 2^nd^ and 3^rd^ trimester.

*Connection to other modules*: The prevalence of late- and early-onset preeclampsia is an input to the Preterm/SGA module, Asphyxia module (which includes stillbirth) and increases the likelihood of preterm, SGA and stillbirth.

*Assumptions:* We make the following assumptions in this module:

- Those with early onset preeclampsia will not have late onset preeclampsia
- Treated preeclampsia incurs no disability

#### Obstructed labour module

*Fig E. Structure of the obstructed labour module.*

*Overview:* The obstructed labour module is a 3-month/1-timestep module that occurs during the childbirth time period and considers the population of pregnant women. DALYs accrue at the end of the module. Pregnant women for whom obstructed labour is imminent are split into those that have a medically necessary c-section and those that do not.

*Outputs:* Outputs for the obstructed labour module are total number of cases of healthy women with respect to obstructed labour, cases of disability due to obstructed labour in referral facility, cases of disability due to obstructed labour (not at a referral facility), maternal deaths due to obstructed labour and DALYs due to obstructed labour.

*Interventions:* Outcomes can be influenced by the ultrasound intervention which targets pregnant women who attend ANC and increases the number of pregnant women with imminent obstructed labour who have delivery at a referral facility (reducing the disability associated with obstructed labour).

*Connection to other modules*: The obstructed labour module receives input for the proportion of women who attend ANC and proportion of women who give birth at a referral facility from the ANC module. The obstructed labour module supplies an input of prevalence of obstructed labour to the hemorrhage module, asphyxia module (which includes stillbirth calculations) and the prevalence of obstructed labour without c-section to the asphyxia module and increases the likelihood of hemorrhage, stillbirth and birth asphyxia incidence.

*Assumptions:* We make the following assumptions in this module:

- All c-sections are medically necessary and carry no disability
- If obstructed labour occurs, birth at a referral facility reduces the probability of mortality by a factor of 2 compared to birth at a different location
- Not all obstructed labours are identifiable by ultrasound

#### Hemorrhage module

*Fig F. Structure of the hemorrhage module.*

*Overview:* The hemorrhage module is a 3-month/1-timestep module that occurs during the childbirth time period and considers the population of pregnant women. DALYs accrue at the end of the module. Pregnant women for whom hemorrhage is imminent are split into those that have a medically necessary c-section and those that do not.

*Outputs:* Outputs for the hemorrhage module are total number of cases of healthy women with respect to hemorrhage, cases of disability due to hemorrhage in referral facility, cases of disability due to hemorrhage (not at a referral facility), maternal deaths due to hemorrhage and DALYs due to hemorrhage.

*Interventions:* Outcomes can be influenced by the ultrasound intervention which targets pregnant women who attend ANC and increases the number of pregnant women with imminent hemorrhage who have delivery at a referral facility (reducing the disability associated with hemorrhage).

*Connection to other modules*: The hemorrhage module receives input for pregnant women at the end of 3^rd^ trimester, proportion of women who attend ANC from the ANC module and prevalence of anemia from the anemia module. The hemorrhage module supplies an input of prevalence of hemorrhage to the anemia module for postpartum bleeding related anemia incidence.

*Assumptions:* We make the following assumptions in this module:

- The mortality rate at a referral facility is half the rate of mortality not at a referral facility

#### Maternal nutrition module

*Fig G. Structure of the maternal nutrition module.*

*Overview:* The maternal nutrition treatment module is a 3-month/single timestep module that occurs during the postpartum time period. It is primary used to determine coverage of undernutrition treatment.

*Outputs:* Outputs for total number of cases of maternal nutrition module are the number of pregnant women continuing to childbirth as well as an implicit nutrition intervention coverage.

*Interventions:* Interventions impact the proportion of pregnant women who are covered by the set of nutrition interventions.

*Connection to other modules*: The maternal nutrition module receives input of pregnant women at the end of 3^rd^ trimester from the ANC module. The maternal nutrition module supplies maternal nutrition intervention coverages to the asphyxia module (for stillbirth risk), stunting module and the preterm/SGA module (for preterm and SGA risk).

*Assumptions:* We make the following assumptions in this module:

- Maternal undernutrition has no maternal disability associated
- Maternal undernutrition does not develop through pregnancy

#### Maternal sepsis module

*Fig H. Structure of the maternal sepsis module.*

*Overview:* The sepsis module is a 3-month/1-timestep module that occurs during the childbirth time period and considers the population of pregnant women. During the postpartum period pregnant women either have sepsis occur or they do not. For pregnant women who do have sepsis they are further split into those that have sepsis at a referral facility and those that do not, with rates of mortality and morbidity due to sepsis determined by the location of birth.

*Outputs:* Outputs for the sepsis module are total number of cases of healthy women with respect to sepsis, cases of disability due to sepsis, cases of death due to sepsis, cases of late maternal death and DALYs due to maternal sepsis.

*Interventions:* Outcomes can be influenced by the azithromycin intervention which targets pregnant women who attend ANC. This reduces the number of pregnant women for whom sepsis occurs.

*Connection to other modules*: Maternal sepsis provides proportion of women with morbidity due to sepsis as an input to the asphyxia module (which includes stillbirth calculation) and increases the likelihood of stillbirth.

*Assumptions:* We make the following assumptions in this module:

- Late maternal death is negligible
- Maternal sepsis only occurs postpartum

#### Preterm/SGA module

*Fig I. Structure of the preterm and SGA module.*

*Overview:* The preterm/SGA module is a 3-month/1-timestep module that occurs during the childbirth time-period and considers the population of neonates. During the childbirth period neonates are split into those who are preterm and/or SGA and those that are healthy with respect to both preterm and SGA. Those who have preterm and/or SGA are further split into those who were born in a referral facility and those that were not. Those born at a referral facility have an increased probability of the condition without any disability.

*Outputs:* Outputs for the preterm/SGA module are total number of cases of healthy with respect to preterm/SGA, cases of Preterm and/or SGA without disability, cases of disability due to preterm and/or SGA, cases of mortality due to preterm and/or SGA and DALYs due to preterm/SGA.

*Interventions:* Outcomes can be influenced by the ultrasound product which implicitly increases the number of births at a referral facility due to likely preterm birth.

*Connection to other modules*: The preterm/SGA module receives inputs from the maternal undernutrition and anemia modules, which contribute to the prevalence of preterm and SGA. The module provides input for the RDS, stunting and neonatal sepsis modules and contributes to the likelihood of RDS, stunting and neonatal sepsis occurring.

*Assumptions:* We make the following assumptions in this module:

- The risk of mortality at a referral facility is half the risk elsewhere
- This is the population of preterm births that do not result in RDS
- Morbidity with Preterm & SGA has the same adverse outcomes as Preterm or SGA.

#### Respiratory distress syndrome module

 *Fig J. Structure of the respiratory distress syndrome module.*

*Overview:* The RDS module is a 3-month/1-timestep module that occurs during the neonatal time-period and considers the population of neonates. Neonates are split into those who are preterm and those that are healthy with respect to both preterm and SGA. RDS will only occur for those that are preterm. For those neonates in whom RDS occurs, outcomes are determined by the severity of preterm and whether or not they are delivered at a referral facility. Probability of mortality is higher in those with birth at <28 weeks gestational age and/or those with delivery not at a referral facility. All cases of RDS without mortality incur a disability.

*Outputs:* Outputs for RDS module are total number of cases of healthy with respect to RDS, cases of disability due to RDS, cases of mortality due to RDS and DALYs due to RDS.

*Interventions:* Outcomes can be influenced by the ultrasound product which implicitly increases the number of births at a referral facility due to likely preterm birth. Although in this iteration of the model no intervention directly targets those with RDS, theoretically an intervention that reduces the likelihood of RDS occurring could be targeted at those neonates with identified preterm birth.

*Connection to other modules*: The RDS module receives inputs of prevalence of preterm from the preterm/SGA module which contribute to the incidence of RDS.

*Assumptions:* We make the following assumptions in this module:

- All cases of RDS incur disability (with the disability weight an average of the severity of outcomes)
- The identification of preterm allows for early treatment of RDS

#### Neonatal sepsis module

*Fig K. Structure of the neonatal sepsis module.*

*Overview:* Neonatal sepsis module is a 3-month/1-timestep module that occurs during the neonatal time-period and considers the population of neonates. Neonates are split into those who are preterm and or SGA and those that are healthy with respect to both preterm and SGA. Those who have preterm and/or SGA are more likely to develop neonatal sepsis, and only cases of identified preterm and/or SGA are treated (note: this iteration of the model has no direct neonatal sepsis treatment).

*Outputs:* Outputs for neonatal sepsis module are healthy with respect to neonatal sepsis, disability due to sepsis and mortality due to sepsis.

*Interventions:* Outcomes can be influenced by the ultrasound product which implicitly increases the number of births at a referral facility due to likely preterm birth. Although in this iteration of the model no intervention directly targets those with neonatal sepsis, theoretically an intervention that reduces the likelihood of neonatal sepsis occurring could be targeted at those neonates with identified preterm birth.

*Connection to other modules*: The neonatal sepsis module receives inputs of prevalence of preterm birth from the preterm/SGA module.

*Assumptions:* We make the following assumptions in this module:

- We approximate a single survival rate for all neonates
- In general only identified preterm births are indicated for treatment

#### Asphyxia module

*Fig L. Structure of the asphyxia module.*

*Overview:* The birth asphyxia module is a 3-month/1-timestep module that occurs during the neonatal time-period and considers the population of neonates. Neonates are split into those for whom birth asphyxia occurs. Those with birth asphyxia at a referral facility have less severe outcomes than those who have birth asphyxia at a different location. Birth asphyxia can result in mortality or morbidity due to encephalopathy.

*Outputs:* Outputs for the birth asphyxia module are healthy with respect to birth asphyxia, disability due to encephalopathy, less severe disability due to encephalopathy, birth asphyxia without encephalopathy and mortality due to encephalopathy.

*Interventions:* Outcomes can be influenced by the ultrasound product which implicitly increases the number of births at a referral facility due to obstructed labour (which is a risk factor for birth asphyxia/encephalopathy.)

*Connection to other modules*: Birth asphyxia receives input of obstructed labour without c-section from the obstructed labour module.

*Assumptions:* We make the following assumptions in this module:

- Treatment averts outcomes by 1 severity level
- The current iteration of the model has no birth asphyxia without encephalopathy
- Disability due to encephalopathy are equally split into ‘disability’ and ‘lesser disability’

#### Stunting module

*Fig M. Structure of the stunting module.*

*Overview:* The stunting module is a 3-month/1-timestep module that occurs during the neonatal time-period and considers the population of surviving neonates. Neonates either have stunting occur or they do not.

*Outputs:* Outputs for stunting are total number of healthy with respect to stunting, disability due to stunting and DALYs due to stunting.

*Interventions:* This iteration of the model has no interventions directly targeting neonates with stunting.

*Connection to other modules*: The stunting module receives inputs of prevalence of preterm and SGA from the preterm/SGA module.

*Assumptions:* We make the following assumptions in this module:

- Stunting produces no mortality
- Any stunting produces a disability

#### Other neonatal conditions module

*Fig N. Structure of the other neonatal conditions module.*

*Overview:* The other neonatal conditions module is a 3-month/1-timestep module that occurs during the neonatal time-period and considers the population of surviving neonates. Other neonatal conditions are the set of conditions that produce morbidity and mortality, but are not considered elsewhere in the model. Neonates either have a condition occur or they do not. If a condition occurs they are either surviving or a mortality occurs. If they are surviving the condition occurs either with or without a disability.

*Outputs:* Outputs for other neonatal conditions are total number of healthy with respect to other neonatal conditions, other neonatal condition occurring without disability, disabilities due to other neonatal condition, mortalities due to other neonatal condition and DALYs due to other neonatal conditions.

*Interventions:* This iteration of the model has no interventions directly targeting neonates with other neonatal conditions.

*Connection to other modules*: The other neonatal conditions does not receive or provide inputs to other modules

#### Wasting module

*Fig O. Structure of the wasting module.*

*Overview:* The wasting module is a 5 year/3-timestep module that occurs from 8 days to 5 years post birth and considers the population of neonates and children. In the first time period from 8 days to 5-months post-birth neonates are either underweight or healthy (but at risk) of wasting. If the neonate/child is underweight and it is not treated either a morbidity or mortality due to underweight occurs. For surviving children in the 6-18 month time period, if wasting occurs the outcomes are determined by the severity of wasting and the location of the wasting treatment (hospitalized or community based). For surviving neonates in the 19-59 month time period the same pathways as in the 6-18 month time period are repeated.

*Outputs:* Outputs for the wasting module are number of mortalities due to underweight (8 days – 5 months), number of disabilities due to underweight (8 days to 5 months), number of mortalities due to wasting (6 – 18 months), number of disabilities due to wasting (6 – 18 months), number of mortalities due to wasting (19 – 59 months), number of disabilities due to wasting (19 – 59 months), number of healthy with respect to wasting and total DALYs due to wasting.

*Interventions:* Outcomes can be impacted by the SOC RUTF intervention which targets children with wasting both at community health centres or at hospitals.

*Connection to other modules*: The wasting module receives inputs of prevalence of preterm and SGA from the preterm/SGA module.

*Assumptions:* We make the following assumptions in this module:

- Separate populations for 6-18 months and 19-59 months are required for RUTF intervention to target different populations
- The proportion of neonates for whom underweight was not calibrated to DALYs or cases of wasting – the data source for proportion of neonates who are underweight was used.
- Only 1 wasting incident can occur per person per time step

### Interaction between modules

#### Impact on relative risks

A key aspect of this model is the ability for the prevalence of a condition in one module to affect the relative risk of conditions in other modules. This is done by including the upstream condition in the calculation for the incidence of a downstream condition. For example, the prevalence of condition A, which has risk factors of prevalence of condition B and prevalence of condition C is:

Prev(A) = Prev(A_0_) * (1+Prev(B)*(RR_B_ – 1)) * (1+Prev(C)*(RR_C_ – 1))

Where Prev(A_0_) is the proportion of the population that would have condition A in the absence of any risk factors (note: this is a theoretical quantity that is calibrated), Prev(B) and Prev(C) are the prevalence of conditions B and C, and RR_B_ and RR_C_ are the relative risk of conditions B and C on condition A, respectively.

This formula can be extended to more than two risk factor conditions. A full listing of risk factors included in the model is provided in Table 2.

Table H. List of cross-module risk factors included in the model

| **Condition** | **Risk factor** |
| --- | --- |
| Anemia | Hemorrhage |
| Hemorrhage | Prevalence of anemia (severe and non-severe) |
| Asphyxia | Prevalence of maternal sepsis |
|  | Prevalence of early and late onset preeclampsia |
| Neonatal sepsis | Prevalence of preterm |
|  | Prevalence of maternal sepsis |
| Preterm/SGA | Prevalence of anemia |
|  | Prevalence of early and late onset preeclampsia |
| RDS | Prevalence of preterm |
| Stunting | Prevalence of preterm |
|  | Prevalence of SGA |
| Wasting | Prevalence of SGA |
|  | Prevalence of preterm |

#### Avoiding double counting of death

Deaths are calculated individually by each module in the model. However, in many cases we are interested in the total number of deaths across all causes for a given combination of interventions. Therefore, we need to account for the overlap in deaths across modules, where the same person may have been assigned mortality in more than one module. This correction is applied after running the model to calculate an adjusted total number of deaths.

Suppose the model reports X people died of condition A, Y people died of condition B, and Z people died of condition C, where the conditions are all in separate modules in the model. To report the total number of deaths, we avoid this double counting by calculating the total number of deaths based on the overall probability of survival, with

N_deaths_ = 1-(1-X)*(1-Y)*(1-Z)

We can also report on the number of people who died of each condition in isolation (e.g., with condition A only). This is done by assuming that conditions are uncorrelated/independent and then removing the overlap with

N_A_ = X * (1-Y)*(1-Z)

N_B_ = Y * (1-X)*(1-Z)

N_C_ = Z * (1-Y)*(1-Z)

where N_A._ N_B_ and N_C_ are the number of people who died of only conditions A, B, and C, respectively. Note that for conditions with strong correlations (e.g., preterm birth and SGA) the conditions were included in the same module so that the correlations could be explicitly accounted for. In the case of people assigned mortality due to multiple conditions, it is ambiguous which condition was the primary cause of death, hence they are excluded from the cause-specific mortalities. However, due to the relatively low prevalence and mortality rate for the conditions included in this study, the number of people in this category is typically negligible.

An additional source of possible double counting is if maternal death or pregnancy loss occurs prior to childbirth. The model tracks continuing pregnancies at each trimester – in the event of pregnancy loss, those pregnancies are removed immediately thereby changing eligibility for interventions later in the pregnancy and changing the number of live births.

1.	Global Burden of Disease Collaborative Network. Global Burden of Disease Study 2017 (GBD 2017) Population and Fertility 1950-2017. Seattle, United States of America: Institute for Health Metrics and Evaluation (IHME), 2018; 2018.

2.	Global Burden of Disease Collaborative Network. Global Burden of Disease Study 2017 (GBD 2017) Incidence, Prevalence, and Years Lived with Disability 1990-2017. Seattle, United States of America: Institute for Health Metrics and Evaluation (IHME); 2018.

3.	Global Burden of Disease Collaborative Network. Global Burden of Disease Study 2017 (GBD 2017) Cause-Specific Mortality 1980-2017. Seattle, United States of America: Institute for Health Metrics and Evaluation (IHME); 2018.

4.	Global Burden of Disease Collaborative Network. Global Burden of Disease Study 2019 (GBD 2019) Burden by Risk 1990-2019. Seattle, United States of America: Institute for Health Metrics and Evaluation (IHME); 2020.

5.	Global Burden of Disease Collaborative Network. Global Burden of Disease Study 2019 (GBD 2019) Life Expectancy and Healthy Life Expectancy 1990-2019. Seattle, United States of America: Institute for Health Metrics and Evaluation (IHME); 2020.

6.	Fall CH, Sachdev HS, Osmond C, Restrepo-Mendez MC, Victora C, Martorell R, et al. Association between maternal age at childbirth and child and adult outcomes in the offspring: a prospective study in five low-income and middle-income countries (COHORTS collaboration). Lancet Glob Health. 2015;3(7):e366-77.

7.	Global Burden of Disease Collaborative Network. Global Burden of Disease Study 2019 (GBD 2019) Healthcare Access and Quality Index 1990-2019. Seattle, United States of America: Institute for Health Metrics and Evaluation (IHME); 2020.

8.	Alliance for M, Newborn Health Improvement mortality study g. Population-based rates, timing, and causes of maternal deaths, stillbirths, and neonatal deaths in south Asia and sub-Saharan Africa: a multi-country prospective cohort study. Lancet Glob Health. 2018;6(12):e1297-e308.

9.	Pregnancy Risk IS, and Measurement Alliance. <https://prisma-mnh.org/> [

10.	Ehwerhemuepha L, Carlson K, Moog R, Bondurant B, Akridge C, Moreno T, et al. Cerner real-world data (CRWD) - A de-identified multicenter electronic health records database. Data in Brief. 2022;42:108120.

11.	Global Burden of Disease Collaborative Network. Global Burden of Disease Study 2019 (GBD 2019) Disease and Injury Burden 1990-2019. Seattle, United States of America: Institute for Health Metrics and Evaluation (IHME); 2020.

12.	Milman N. Postpartum anemia I: definition, prevalence, causes, and consequences. Annals of Hematology. 2011;90:1247–53.

13.	Global Burden of Disease Collaborative Network. Global Burden of Disease Study 2017 (GBD 2017) Disability Weights. Seattle, United States of America: Institute for Health Metrics and Evaluation (IHME); 2018.

14.	Naz S, Jaffar A, Yazdani N, Kashif M, Hussain Z, Khan U, et al. Cohort profile: the Pregnancy Risk Infant Surveillance and Measurement Alliance (PRISMA) – Pakistan. 2023;13(12):e078222.

15.	Magee LA, von Dadelszen P. State-of-the-Art Diagnosis and Treatment of Hypertension in Pregnancy. Mayo Clin Proc. 2018;93(11):1664-77.

16.	Abalos E, Cuesta C, Grosso AL, Chou D, Say L. Global and regional estimates of preeclampsia and eclampsia: a systematic review. Eur J Obstet Gynecol Reprod Biol. 2013;170(1):1-7.

17.	Global Burden of Disease Collaborative Network. Global Burden of Disease Study 2017 (GBD 2017) Disability-Adjusted Life Years and Healthy Life Expectancy 1990-2017. Seattle, United States of America: Institute for Health Metrics and Evaluation (IHME); 2018.

18.	Beck C, Gallagher K, Taylor LA, Goldstein JA, Mithal LB, Gernand AD. Chorioamnionitis and Risk for Maternal and Neonatal Sepsis: A Systematic Review and Meta-analysis. Obstet Gynecol. 2021;137(6):1007-22.

19.	Erchick DJ, Hazel EA, Katz J, Lee ACC, Diaz M, Wu LSF, et al. Vulnerable newborn types: analysis of subnational, population-based birth cohorts for 541 285 live births in 23 countries, 2000-2021. BJOG. 2023.

20.	Kozuki N, Lee AC, Katz J, Child Health Epidemiology Reference G. Moderate to severe, but not mild, maternal anemia is associated with increased risk of small-for-gestational-age outcomes. J Nutr. 2012;142(2):358-62.

21.	Abalos E, Duley L, Steyn DW. Antihypertensive drug therapy for mild to moderate hypertension during pregnancy. Cochrane Database Syst Rev. 2014(2):CD002252.

22.	van Esch JJA, van Heijst, A. F., de Haan, A. F. J., & van der Heijden, O. W. H. Early-onset preeclampsia is associated with perinatal mortality and severe neonatal morbidity. The Journal of Maternal-Fetal &amp; Neonatal Medicine. 2017;30:2789–94.

23.	Bernardes TP, Mol BW, Ravelli ACJ, van den Berg P, Boezen HM, Groen H. Early and late onset pre-eclampsia and small for gestational age risk in subsequent pregnancies. PLoS One. 2020;15(3):e0230483.

24.	Hazel EA, Erchick DJ, Katz J, Lee ACC, Diaz M, Wu LSF, et al. Neonatal mortality risk of vulnerable newborns: A descriptive analysis of subnational, population-based birth cohorts for 238 203 live births in low- and middle-income settings from 2000 to 2017. BJOG. 2023.

25.	Childhood Acute I, Nutrition N. Childhood mortality during and after acute illness in Africa and south Asia: a prospective cohort study. Lancet Glob Health. 2022;10(5):e673-e84.

26.	Mertens A, Benjamin-Chung J, Colford JM, Coyle J, van der Laan MJ, Hubbard AE, et al. Risk factors and impacts of child growth faltering in low- and middle-income countries. 2021:2020.06.09.20127100.

27.	Harmon QE, Huang L, Umbach DM, Klungsoyr K, Engel SM, Magnus P, et al. Risk of fetal death with preeclampsia. Obstet Gynecol. 2015;125(3):628-35.

28.	Christian P, Lee SE, Donahue Angel M, Adair LS, Arifeen SE, Ashorn P, et al. Risk of childhood undernutrition related to small-for-gestational age and preterm birth in low- and middle-income countries. Int J Epidemiol. 2013;42(5):1340-55.

29.	Investigators TM-EN. The MAL-ED Study: A Multinational and Multidisciplinary Approach to Understand the Relationship Between Enteric Pathogens, Malnutrition, Gut Physiology, Physical Growth, Cognitive Development, and Immune Responses in Infants and Children Up to 2 Years of Age in Resource-Poor Environments. Clinical Infectious Diseases. 2014;59(suppl_4):S193-S206.
