# Supplementary material for: A coupled model to value indirect impacts of maternal, newborn and child health interventions
Source: PLOS Glob Public Health. 2026 Nov 6;6(11):e0005507. doi: 10.1371/journal.pgph.0005507 (PMC13742954; doi:10.1371/journal.pgph.0005507)
Supplement: Supplementary file 2 [file pgph.0005507.s002.docx]

# **ISPOR-SMDM Task Force-1 Checklist**

*Caro et al., Task Force-1 Overview (Value in Health, 2012).*

| **#** | **Recommendation** | **Paper location** |
| --- | --- | --- |
| **II. Conceptualizing the Model** | |  |
| **II-1** | The modeling team should consult widely with subject experts and stakeholders to assure that the model represents disease processes appropriately and adequately addresses the decision problem. | Introduction, paragraph 5 (model developed in collaboration with the Bill and Melinda Gates Foundation); Methods - “Model overview and study design,” paragraph 5 (pathway choices developed in consultation with content experts at the Gates Foundation). |
| **II-2** | A clear, written statement of the decision problem, modeling objective, and scope of the model should be developed. This should include: the spectrum of disease considered, perspective of the analysis, target population, alternative interventions, health and other outcomes, and time horizon. | Methods section overall (“Model overview and study design,” “Model outputs,” “Setting,” and “Interventions” subsections collectively define scope). |
| **II-2a** | The scope and structure of the model should be consistent with, and adequate to address, the decision problem/objective and the policy context. | Methods – “Model overview and study design,” paragraphs 1–2. |
| **II-2b** | The perspective of the analysis should be stated and defined. Outcomes modeled in the analysis should be consistent with the stated perspective. Analyses which take a perspective narrower than the societal perspective should report which outcomes are included and which are excluded. | Methods – “Model outputs” paragraph 1 |
| **II-2c** | The target population should be defined in terms of geography, patient characteristics (including co-morbid conditions), and disease stage, each of which should be appropriate to the decision problem. | Methods – “Model overview and study design,” paragraph 3 (trimester tracking); Methods – “Setting” paragraph (three regional LMIC groupings); Table 1, “Wasting” row (age-band timesteps). |
| **II-2d** | Health outcomes modeled in the analysis, which may be measured as events, cases of disease, deaths, quality-adjusted life-years, disability-adjusted life-years, or other measures important to decision makers and stakeholders, should be directly relevant to the question being asked. | Abstract - Results statement (DALYs averted figures); Introduction, paragraph 5. |
| **II-2e** | Interventions or strategies modeled in the analysis should be clearly defined in terms of frequency, component services (including services that may have preceded the intervention and that would affect its course), dose or intensity, duration, and any variations required for target subgroups. | Table 2 (eligible populations, effect sizes); subgroup variation is assumed absent or captured via coverage; dosage/frequency/duration not considered relevant to this model. |
| **II-3** | Although data are an essential component of a model, the conceptual structure of a model should be driven by the decision problem or research question and not determined by data availability. | Introduction, paragraph 5. |
| **II-3a** | The choice of strategies/comparators crucially affects results, and should be determined by the decision problem and not by data availability or quality. All feasible and practical strategies should be considered. Constraining the range of strategies should be justified. | Methods – “Scenarios design” paragraph 1. |
| **II-3b** | The time horizon of the model should be long enough to capture relevant differences in outcomes across strategies. A lifetime horizon may be required. | Methods – “Scenarios design” paragraph 1 (2024–2040 horizon; scale-up target by 2030). |
| **II-4** | The conceptual representation of the decision problem should be used to identify key uncertainties in model structure where sensitivity analyses could inform the impact of structural choices. For example, where a lifetime horizon is used, the impact of alternative methods of extrapolating beyond the observed data should be explored. | Methods for changing the model structure are described in the supplement. |
| **II-5** | The policy context of the model should be clearly stated. This includes who funded the model, who developed the model, whether the model was developed for a single application or multiple potential applications, and who the policy audience for the modeling work is. | Abstract – “Funding” statement; Introduction, paragraph 5. |
| **II-6** | An explicit process (expert consultations, influence diagrams, concept mapping, or similar method) should be used to convert the conceptualization of the problem into an appropriate model structure to ensure that the model reflects current theory of disease or the process being modeled. | Methods – “Model overview and study design,” paragraph 5. |
| **II-7** | There are often several types of models that are suitable for the decision problem, and versions of each of the three modeling types in the series can be used for the same decision problem. Some problems are more naturally represented in certain modeling types than in others. | Methods – “Model overview and study design,” paragraph 2 (introduces the 15 interconnected modules as deterministic compartmental models). The model is a bespoke network of linked compartmental modules that doesn't map cleanly onto a single technique in the TF-1 series – the closest fit is state-transition (see Section III). |
| **II-8** | Model simplicity is desirable for transparency, ease of validation and description. However, the model should be sufficiently complex to answer the question at a level of detail consistent with the problem being modeled, and to preserve face validity to clinical experts. Greater complexity may be necessary in policy models that are intended to be used for many decision problems. | Methods – “Setting” paragraph; Methods – “Model overview and study design,” paragraph 4. (Discussed via regional aggregation choices and module-level structure.) |
| **III. State-Transition Models** | | |
| **III-1** | If the decision problem can be represented with a manageable number of health states that incorporate all characteristics relevant to the decision problem, including the relevant history, a cohort simulation should be chosen because of its transparency, efficiency, ease of debugging, and ability to conduct specific value of information analyses. If, however, a valid representation of any aspect of the decision problem would lead to an unmanageable number of states, then an individual-level state-transition model is recommended. Validity should not be sacrificed for simplicity. | Abstract (Methods); Methods – “Model overview and study design,” paragraph 1 (compartment-based model with 15 interconnected modules). |
| **III-2** | The strategies being evaluated should be clearly defined. In particular, sequential decisions should not be modeled within the Markov cycle tree but rather be part of the specification of the alternative intervention strategies that precede the Markov tree. | Methods - “Interventions” section (target module, target parameter, effect size, baseline/max coverage); Table 2. |
| **III-3** | The starting cohort should be defined by the demographic and clinical characteristics that affect the transition probabilities or state values (e.g., quality of life and cost). | Methods – “Model overview and study design,” paragraph 3 (women enter the model at pregnancy, tracked by trimester); Methods – “Setting” paragraph (three regional groupings). |
| **III-4** | Specification of states and transitions should generally reflect the biological/theoretical understanding of the disease or condition being modeled. | Introduction, paragraph 3 (anemia–SGA–preterm evidence linkage); Methods – “Model overview and study design,” paragraph 5 (pathway choices developed with content experts, consistent with LiST). |
| **III-5** | States should adequately capture the type of intervention (i.e., prevention, screening, diagnostics, treatment) as well as the intervention’s benefits and harms. | Table 2, “Direct impacts” / “Indirect impacts” columns; Methods “Interventions” section. |
| **III-6** | States need to be homogeneous with respect to both the observed and unobserved (i.e., not known by the decision maker) characteristics that affect transition probabilities. | Methods – “Setting” paragraph (parameters represent the aggregate of LMICs within each region, rather than being disaggregated further). |
| **III-7** | The time horizon for the model should be sufficiently large to capture all health effects and costs relevant to the decision problem. | Methods – “Scenarios design” paragraph (states the 2024–2040 horizon); Discussion – limitations paragraph (horizon not tested against an alternative). |
| **III-8** | Cycle length should be short enough to represent the frequency of clinical events and interventions. | Methods – “Model overview and study design,” paragraph 3 (trimester cycles); Table 1, “Wasting” row (age-band timesteps: 8 days–5 months, 6–18 months, 19–59 months). |
| **III-9** | Components of state-transition models that reflect similar clinical courses should not be recreated but rather should be incorporated once and linked to that structure throughout the model. | Table 1, “Inputs from other modules” column throughout; Discussion, paragraph 6 (model interactions between conditions). |
| **III-10** | Transition probabilities and intervention effects should be derived from the most representative data sources for the decision problem. | Table 2, “Data source” column; Methods – “Interventions” section (analysis-specific), paragraph 2 (non-invasive Hb test and AI ultrasound sourced from expert advice rather than empirical data). |
| **III-11** | All methods and assumptions used to derive transition probabilities and intervention effects should be described. | Methods – “Model parameters” paragraph; Table 2 (per-parameter source documentation); Data described in supplementary material. |
| **III-12** | Parameters relating to the effectiveness of interventions derived from observational studies should be correctly controlled for confounding. Time-varying confounding is of particular concern in estimating intervention effects. | Introduction, paragraph 3 cites observational associations (references 4–9) without discussion of confounding adjustment in Methods. |
| **III-13** | The valuation of intermediate outcomes/states should be justified. | Methods – “Model outputs,” paragraph 1 (outputs converted to DALYs); disability-weight justification in Supplementary material. |
| **III-14** | A half-cycle correction factor should be applied to both costs and effectiveness and should be applied in the first cycle. A half-cycle correction should also be applied in the final cycle for analyses that do not use a lifetime horizon. | Not applicable |
| **III-15** | For certain decision problems, it may be important to report not only the expected value but also the distribution of the outcomes of interest. | Results – Table 3 and Figures 2–3 report point estimates only. |
| **III-16** | The number of individuals modeled in an individual-based simulation should be large enough to generate stable estimates of the expected value of interest. | Population-based, not individual-level simulation. |
| **III-17** | The report should use nontechnical language and clear figures and tables that enhance the understanding of the model to communicate its key structural elements, assumptions, and parameters. | Figure 1 (model structure); Table 1 (module descriptions); Table 2 (intervention definitions). |
| **III-18** | In addition to final outcomes, intermediate outcomes that enhance the understanding and transparency of the model results should also be presented. | Results – Table 3 (direct vs. indirect DALYs by intervention); Figure 2 (reduction in DALYs by condition and scenario); Figure 3 (reduction in key model outcomes). |
| **(scope note)** | Discrete Event Simulation recommendations (IV-1 to IV-17) are not included here. | The MNCH model is a continuous-time deterministic compartmental model. These two full sections are not applicable. |
| **VI. Parameter Estimation and Uncertainty (TF-1)** | | |
| **VI-1** | The systematic examination and reporting of uncertainty are hallmarks of good modeling practice. All modeling studies should therefore include an assessment of uncertainty as it pertains to the decision problem being addressed. | No uncertainty analysis shown, noting this is an example use case of a developed model (not for policy implication.) |
| **VI-2** | The role of the decision maker should be considered when presenting uncertainty analyses. In particular, the description of analytic perspective should include an explicit statement regarding what is assumed about the power of the decision makers to delay or review decisions and to commission or mandate further research. | N/A (Dependent on the specific use case.) |
| **VI-3** | Terminology to describe concepts relating to parameter estimation and representation of uncertainty varies within the medical decision modeling field and in comparison to related fields. Authors should be aware of this and seek to carefully define their use of terminology to avoid potential confusion. | Methods – “Interventions” section (direct/indirect effect terminology is defined); no uncertainty-specific terminology is present since no uncertainty analysis exists. |
| **VI-4** | All decision models will have parameters that need to be estimated. In populating models with parameter estimates, analysts should conform to the broad principles of evidence-based medicine. For example, analysts should: seek to identify and incorporate all relevant evidence, rather than cherry picking the best single source of evidence for that parameter; use best practice methods to avoid potential biases in parameter estimates that might arise (for example, when estimating treatment effectiveness from observational sources); and employ formal evidence syntheses techniques (meta analysis and network meta analysis) as appropriate. | Table 2, “Data source” column (meta-analyses cited for most parameters); Methods – “Interventions” section (analysis-specific), paragraph 2 (non-invasive Hb test sensitivity and AI ultrasound effect size rely on expert opinion, acknowledged as an assumption). |
| **VI-5** | Whether employing deterministic sensitivity analysis methods (point estimate and range) or probabilistic sensitivity analysis (parameterized distribution) the link to the underlying evidence base should be clear. | Table 2, “Data source” column. |
| **VI-6** | While completely arbitrary analyses, such as the presentation of the effect on model outputs of varying each input parameter by ±50%, can be used as a measure of sensitivity, such analyses should not be used to represent uncertainty. | Arbitrary variation not included. |
| **VI-7** | Analysts should give consideration to using commonly adopted standards from statistics for point estimate and interval estimation for input parameters, such as 95% confidence intervals, or distributions based on agreed statistical methods for a given estimation problem. Where departures from these standards are deemed necessary (or where no such standard exists for a given estimation problem), these should be justified. | Confidence intervals not reported; point estimates used since this is an example use case of a developed model (not for policy implication.) |
| **VI-8** | When there is very little information on a parameter, analysts should adopt a conservative approach such that the absence of evidence is reflected in a very broad range of possible estimates. On no account should parameters be excluded from a sensitivity analysis on the grounds that ‘there is not enough information from which to estimate uncertainty’. | N/A |
| **VI-9** | In choosing distributional forms for parameters in a probabilistic sensitivity analysis, favor should be given to continuous distributions that provide a realistic portrayal of uncertainty over the theoretical range of the parameter of interest. Hence careful consideration should be given to whether distributions like the triangular should have any role in a probabilistic sensitivity analysis. | N/A |
| **VI-10** | Correlation among parameters should be considered. Jointly estimated parameters, such as those from a regression analysis, will have direct evidence on correlation which should be reflected in the analysis. Independently estimated parameters will have no such evidence, but this should not necessarily lead to an assumption of independence. Possible approaches are 1) to include a correlation coefficient as a parameter to the model where concern exists that an unknown correlation between parameters could be important, or 2) to reparameterize the model so that that the uncertain parameters can be reasonably assumed to be independent. | N/A |
| **VI-11** | Where uncertainties in structural assumptions were identified in the process of conceptualizing and building a model, those assumptions should be tested in a sensitivity analysis. Consideration should be given to opportunities to parameterize these uncertainties for ease of testing. Where it is not possible to perform structural sensitivity analysis it is nevertheless important that analysts be aware of the potential for this form of uncertainty to be at least as important as parameter uncertainty for the decision maker. | Discussion - limitations paragraph (structural assumptions discussed narratively as limitations; not formally tested in sensitivity analysis). |
| **VI-12** | Uncertainty analyses can be either deterministic or probabilistic, and often it is appropriate to report aspects of both types within a single evaluation. Tornado diagrams, threshold plots, or simple statements of threshold parameter values, are all appropriate ways of reporting results from deterministic sensitivity analyses. | N/A |
| **VI-13** | When additional assumptions or parameter values are introduced for purposes of uncertainty analyses, such as distributional parameters for probabilistic sensitivity analyses, or parameter ranges for deterministic sensitivity analyses, these values should be disclosed and justified. Technical appendices are often appropriate for this purpose. | N/A |
| **VI-14** | When model calibration is used to derive parameters, uncertainty around the calibrated values should also be reported, and this uncertainty should be reflected in either deterministic or probabilistic sensitivity analyses, or both. | Methods - “Model calibration” paragraph (method described qualitatively; no calibration uncertainty reported). |
| **VI-15** | When the purpose of a probabilistic sensitivity analysis is to guide decisions about acquisition of information to reduce uncertainty, results should be presented in terms of expected value of information. | N/A |
| **VI-16** | For economic studies, when a probabilistic sensitivity analysis is performed without an accompanying expected value of information analysis, options for presenting results include cost-effectiveness acceptability curves (CEACs), and distributions of net monetary benefit or net health benefit. When more than two comparators are involved, CEACs for each comparator should be plotted on the same graph. | N/A |
| **VII. Transparency and Validation** | | |
| **VII-1** | Every model should have non-technical documentation that is freely accessible to any interested reader. At a minimum it should describe in non-technical terms the type of model and intended applications; funding sources; structure of the model; inputs, outputs, other components that determine the model’s function, and their relationships; data sources; validation methods and results; and limitations. | Methods, Results, and Discussion sections collectively. |
| **VII-2** | Every model should have technical documentation, written in sufficient detail to enable a reader with the necessary expertise to evaluate the model and potentially reproduce it. The technical documentation should be made available openly or under agreements that protect intellectual property, at the discretion of the modelers. | Supplementary Material (framework, databook, and data sources). |
| **VII-3** | Validation of a model should include an evaluation of face validity of the structure, evidence, problem formulation, and results of the model. A description of the process used to evaluate face validity should be made available on request. Evaluation of face validity should be made by people who have expertise in the problem area, but are impartial to the results of an analysis. If face validation raises questions about a model, these issues should be discussed by the modelers in their report of an analysis. | Methods – “Model overview and study design,” paragraph 5 (validity assessed by content and modelling experts). |
| **VII-4** | Models should be subjected to rigorous verification. The verification methods should be described in the non-technical documentation of the model. The pertinent results of verification should be made available on request. | Methods - “Model calibration” section. |
| **VII-5** | Modelers should search for previously published modeling analyses of the same or similar problems and discuss insights gained from similarities and differences in results. | Discussion, paragraph 5 (comparison to LiST, EQUIST, Optima, MANDATE, HEAT/HEAT-Plus, REDUCE-ALIVE). |
| **VII-6** | Builders of models should have a formal process for conducting external validation that includes: systematic identification of suitable data sources; justification of the selection; specification of whether a data source is dependent, partially dependent, or independent; and description of which parts of the model are evaluated by each data source. Simulation of each data source and comparison of results. Quantitative measures of how well the model’s results match the outcomes observed in the data source. | Results – “Validation” paragraph (qualitative comparison to Ward et al. and LiST); Supplementary material. |
| **VII-7** | Comparison of results should include descriptions of: data source; set up of the simulation; discrepancies between the data source and simulation setup, and implications of the discrepancies; comparisons of simulation results with observed results; discussion of discrepancies between simulation results and observed results; sensitivity analyses. | Results – “Validation” paragraph (discrepancies in scope/timeframe discussed); no accompanying sensitivity analysis, noting this is a model-introduction paper rather than a full economic evaluation. |
| **VII-8** | Modelers should make available on request a description of the external validation process and results. | Discussion, paragraph 5 (comparison to previous models). Resources to recreate results and analyses included in supplementary materials. |
| **VII-9** | Modelers should identify parts of a model that cannot be validated because of lack of suitable data sources, and describe how uncertainty about those parts is addressed. | Discussion – limitations paragraph (validation constraints discussed narratively). |
| **VII-10** | For multi-application models, modelers should describe criteria for determining when validations should be repeated and/or expanded. | N/A – dependent on the specific use case for the model. |
| **VII-11** | When feasible with respect to the decision being addressed and the availability of a future data source, a model should be tested for its prediction of future events. Builders of multiple-use models should seek opportunities to conduct predictive validations as part of their overall validation process. | Not yet feasible – model is prospective (2024–2040); no future data yet exists to test against. |
